# Differential Reliance on Lipid Metabolism as a Salvage Pathway Underlies Functional Differences of T Cell Subsets in Poor Nutrient Environments

**DOI:** 10.1016/j.celrep.2018.03.084

**Published:** 2018-04-17

**Authors:** Christopher Ecker, Lili Guo, Stefana Voicu, Luis Gil-de-Gómez, Andrew Medvec, Luis Cortina, Jackie Pajda, Melanie Andolina, Maria Torres-Castillo, Jennifer L. Donato, Sarya Mansour, Evan R. Zynda, Pei-Yi Lin, Angel Varela-Rohena, Ian A. Blair, James L. Riley

**Affiliations:** 1 Department of Microbiology and Center for Cellular Immunotherapies, Perelman School of Medicine, University of Pennsylvania, Philadelphia, PA 19104, USA; 2 Department of Pharmacology, Perelman School of Medicine, University of Pennsylvania, Philadelphia, PA 19104, USA; 3 Gibco BioProduction Cell Culture and Cell Therapy, Thermo Fisher Scientific, 3175 Staley Road, Grand Island, NY 14072, USA

## Abstract

T cells compete with malignant cells for limited nutrients within the solid tumor microenvironment. We found that effector memory CD4 T cells respond distinctly from other T cell subsets to limiting glucose and can maintain high levels of interferon-γ (IFN-γ) production in a nutrient-poor environment. Unlike naive (T_N_) or central memory T (T_CM_) cells, effector memory T (T_EM_) cells fail to upregulate fatty acid synthesis, oxidative phosphorylation, and reductive glutaminolysis in limiting glucose. Interference of fatty acid synthesis in naive T cells dramatically upregulates IFN-γ, while increasing exogenous lipids in media inhibits production of IFN-γ by all subsets, suggesting that relative ratio of fatty acid metabolism to glycolysis is a direct predictor of T cell effector activity. Together, these data suggest that effector memory T cells are programmed to have limited ability to synthesize and metabolize fatty acids, which allows them to maintain T cell function in nutrient-depleted microenvironments.

## In Brief

Ecker et al. distinguish unique metabolic and functional properties of naive and memory T cell subsets during glucose limitation. During glucose starvation, T cells begin to differentially rely on fatty acid synthesis and glutamine utilization to survive. Unexpectedly, reliance on fatty acid synthesis alters the ability to produce IFN-γ.

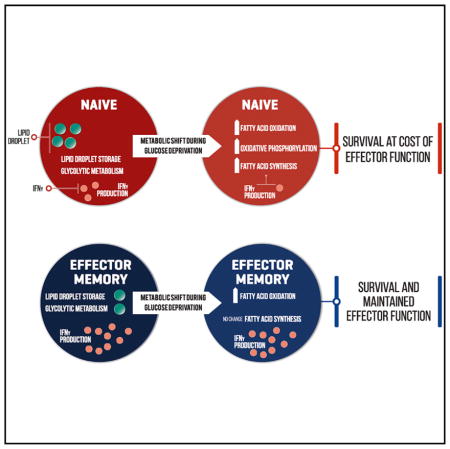

## INTRODUCTION

T cell responses against tumor are often blunted by the recruitment of suppressive immune cells, immune checkpoint blockade, exhaustion, and competition for vital nutrients (Chang et al., 2015a; Dunn et al., 2002; Jacobs et al., 2008; Moon et al., 2014; Sukumar et al., 2015). Both tumor cells and activated effector T cells rely heavily on glycolysis, and recent work has demonstrated that tumor cells are able to outcompete T cells for scarce glucose (Chang et al., 2015a; Ho et al., 2015). The most well-characterized defect in effector response due to poor glucose availability is the pronounced reduction in interferon-γ (IFN-γ) production following activation of T cells (Cham and Gajewski, 2005; Chang et al., 2013; Siska et al., 2016). Two mechanisms behind glucose-mediated IFN-γ downregulation have been proposed: (1) GAPDH, when not involved in glycolysis, binds to the 3′ UTR of IFN-γ and prevents IFN-γ RNA from being translated (Chang et al., 2013); and (2) the steady-state levels of cytosolic acetyl-coenzyme A (acetyl-CoA) is reduced in limiting glucose, reducing histone acetylation at sensitive sites like the IFN-γ locus and thus lowering production of IFN-γ (Peng et al., 2016). However, it is unclear whether either of these two mechanisms are operative and equally active in all T cell subsets.

Most data studying T cell responses in the presence of limiting glucose have used cells which are largely naive T (T_N_) cells rather than human effector memory T (T_EM_) cells, which are the population enriched in the tumor microenvironment (Beura et al., 2016). T cell subsets have remarkably different proliferative capacities, trafficking patterns, and effector capabilities (Sallusto et al., 1999). T_EM_ cells are defined by the lack of lymphatic homing markers such as CCR7 and CD62-L and loss of the co-stimulatory molecule CD27. T_EM_ cells do not proliferate well relative to naive or central memory T (T_CM_) cells but have enhanced effector functions such as cytotoxic potential and effector cytokine production.

Few studies have examined the metabolism of human T_EM_ cells, because they are difficult to culture and scarce in the peripheral blood of healthy donors. The studies that have been performed have demonstrated that T_EM_ cells in hypoxia have a survival advantage and are uniquely adapted to produce IFN-γ rapidly (Dimeloe et al., 2016; Gubser et al., 2013; Xu et al., 2016). Human T_EM_ cells are the most common T cell to reside in the tumor microenvironment and other inflamed environments (Farber et al., 2014; Pagès et al., 2005; Thome et al., 2014). Inflammation often disrupts the vasculature and can induce hypoxia and deprive cells of valuable nutrients in the inflamed tissue (Eltzschig and Carmeliet, 2011). Thus, T_EM_ cells are often forced to function in environments that are nutrient deprived. We hypothesized that because T_EM_ cells must function in nutrient deprived environments, they may have unique metabolic mechanisms to adapt compared to T_N_ or T_CM_ cells. Recent work has shown that fatty acid oxidation and synthesis is essential for survival, growth, and metastatic expansion of pancreatic cancer and other cancer cells *in vivo* (Ricciardi et al., 2015; Samudio et al., 2010; Svensson et al., 2016). We speculated that if pancreatic cancer cells became reliant on fatty acids, then T cells found in the same limited nutrient environment might rely on a similar metabolism.

Here, we demonstrate that, like many cancer cells, when T_N_ and T_CM_ cells are starved of glucose, they augment fatty acid metabolism, which drives oxidative phosphorylation and allows these two T cell subsets to survive in limited glucose. This increased reliance on fatty acid oxidation and synthesis correlated with reduced IFN-γ expression upon T cell activation. In contrast, T_EM_ cells did not upregulate fatty acid synthesis and could maintain high levels of IFN-γ production in low glucose upon T cell activation. Together, these data suggest that T_EM_ cells are programmed to have limited ability to synthesize and metabolize fatty acids, and as a result, T_EM_ cells maintain functionality in limiting glucose conditions.

## RESULTS

### Generation of a Chemically Defined, Customizable Medium that Can Expand Human T Cell Subsets in the Absence of Serum

**Figure 1:**
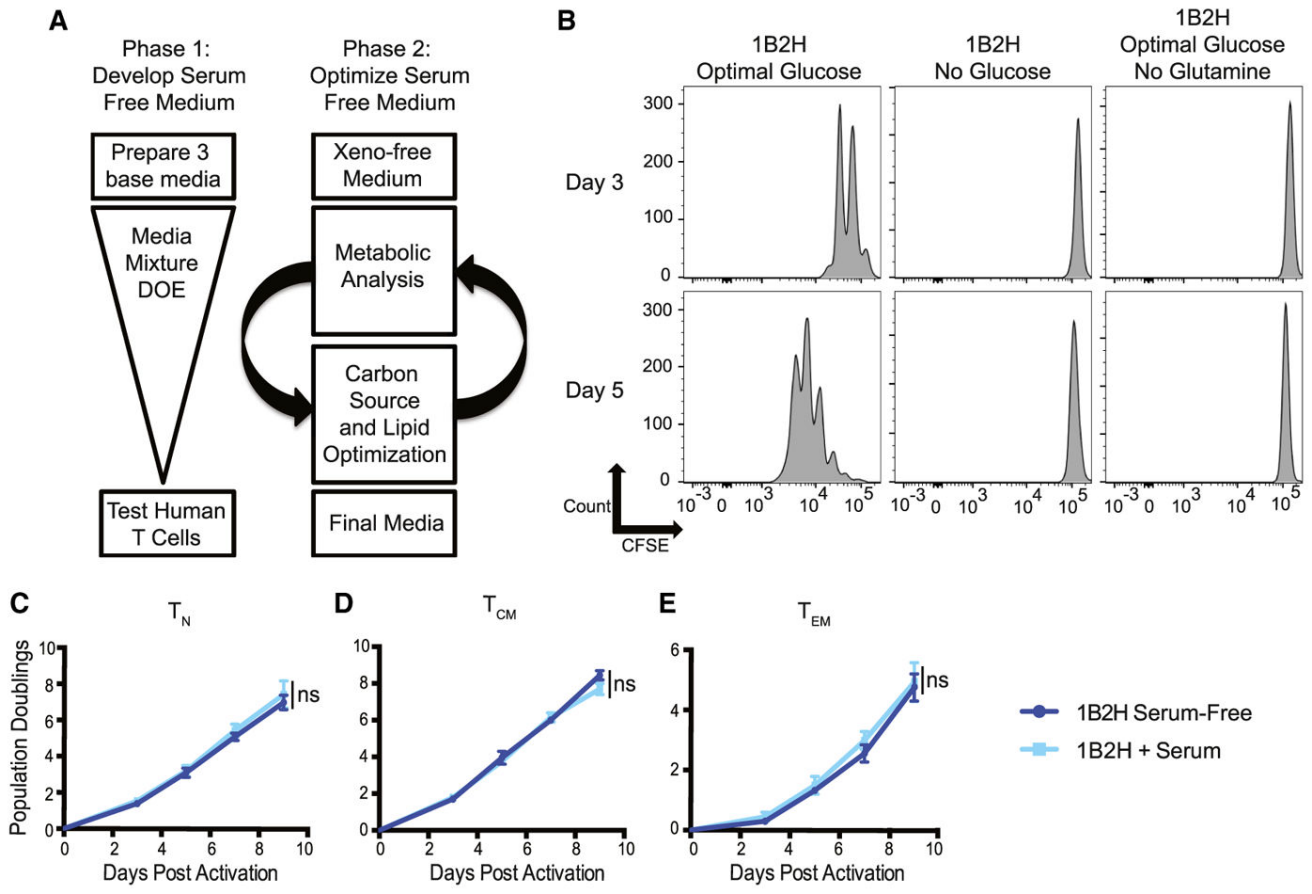
Generation of a Chemically Defined, Customizable Media that Can Expand Human T Cell Subsets in the Absence of Serum (A) The first phase of generating a serum-free medium that can expand all human T cell subsets consisted of creating 10 prototype media by mixing different ratios of 3 base media that contain different concentrations of amino acids, vitamins, trace elements, antioxidants, metal ions, polyamines, and lipids. These prototype media were tested for their ability to expand activated primary human T cells using anti-CD3/CD28-coated beads and reiterated with a design of experiments (DOE) statistical quadratic model through Design-Expert 9.0.1 software with a desired response to maximally expand human T cells without serum supplementation. Phase 2 consisted of eliminating xenogeneic components, examining metabolites consumed in serum-supplemented X-VIVO-15 medium and prototype media from phase 1, and modifying media so that concentrations of metabolites are maintained upon feeding of activated T cells. The final phase focused on optimizing carbon sources, lipid concentrations, lentiviral transduction efficiency, and cytokine production post-activation on activated human T cells. (B) Total CD4 T cells were labeled with carboxyfluorescein succinimidyl ester (CFSE) and activated by anti-CD3/CD28-coated beads in 1B2H medium containing optimal glucose, no glucose, or optimal glucose without glutamine. T cell proliferation was measured by CFSE dilution by flow cytometry. Data are representative of 3 independent experiments. (C–E) Primary human CD4 T cells were sort-purified into T_N_ (CD25^−^CD45RA^+^CCR7^+^CD27^+^) (C), T_CM_ (CD25^−^CD45RO^+^CCR7^+^CD27^+^) (D), and T_EM_ cells (CD25^−^CD45RO^+^CCR7^−^CD27^−^) (E) and stimulated with anti-CD3/CD28-coated beads in 1B2H medium with or without 5% human serum (see Figure S2 for gating strategy). Cell expansion was monitored by Coulter counter on the indicated days. Data are representative of 2–3 donors and independent experiments. *p < 0.05, **p < 0.01, paired two-tailed Student’s t test on day 9 population doublings; ns, not significant.

To date, studies that have examined the effects of nutrient availability on T cell function *in vitro* have relied on media supplemented with dialyzed serum (Blagih et al., 2015; Chang et al., 2013; Keppel et al., 2015). Serum is ill-defined, making characterization of key nutrients challenging. Moreover, dialysis of serum is nonspecific. To overcome these limitations, we sought to create a chemically defined medium that could expand all human T cell subsets without supplementation of human serum. Three different basal media that contained selected groups of components, including fatty acids, metal elements, polyamines, and antioxidants, were generated for the study. These media were used alone or mixed at different ratios to generate ten media for the design of experiments (DOE) screen (Table S1A). T cells from seven healthy donors were activated with anti-CD3/CD28-coated beads, and their expansion rates were monitored in each of the ten media (Figure S1). Statistical analysis, harmonization to eliminate components of xenogeneic origin, and rational approaches based on spent media samples were used to further optimize the media (Figures 1A, S1A, and S1B; Tables S1B and S1C). Lastly, using a range of concentrations of glucose, galactose, and lipids, the carbon source supplied to the T cells was optimized (Figure S1C and S1D; Tables S1A–S1C) and compared to that of medium actively being used in clinical trials of adoptive T cell therapy, X-VIVO 15 media supplemented with 5% human serum. While physiological glucose concentration in human blood is ~5 mM, we found that the static optimal glucose concentration that allowed maximal T cell expansion is 35 mM (Table S2). To ensure that our medium, called 1B2H, could be customized to study T cell metabolism, we generated 1B2H variants that were glucose or glutamine free. We found that T cells could not divide in media free of glucose or glutamine, demonstrating that our media contained no nutrients that could substitute for either glucose or glutamine.

To examine how well human T cell subsets grew in our created medium in the absence of serum, we sorted T_N_, T_CM_, and T_EM_ CD4 T cells and compared expansion in our created medium (1B2H) supplemented with or without human serum. We found that all subsets could grow equally well in 1B2H medium with or without serum supplementation (Figures 1C–1E), and this level of T cell expansion is consistent with serum-supplemented commercial media that is currently being used for adoptive T cell therapy (Medvec et al., 2018). Thus, 1B2H is a bona fide serum-free, customizable medium that can facilitate the study of metabolic differences between human T cell subsets.

### Effector Memory T Cells Are Resistant to Glucose-Mediated IFN-γ Suppression

**Figure 2:**
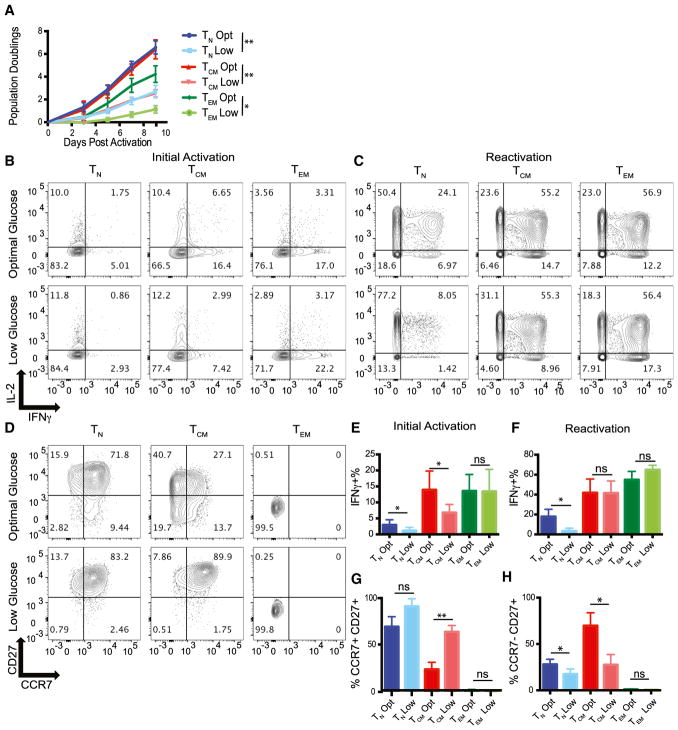
Effector Memory T Cells Are Resistant to Glucose-Mediated IFN-γ Suppression (A) The indicated subsets were stimulated with anti-CD3/CD28 coated beads in the presence of optimal (35 mM) or low (0.35 mM) glucose. Cell expansion was monitored by Coulter counter on the indicated days. Statistics were performed on day 9 population doublings. (B and C) T cell subsets that were expanded for 24 hr (B) with anti-CD3/CD28-coated beads or expanded for 9–11 days (C) with anti-CD3/CD28-coated beads before IFN-γ/IL-2 production was measured after PMA/ionomycin treatment. (D) CCR7 and CD27 expression measured on T cell subsets described in (A) 7 days after T cell expansion. (E and F) IFN-γ production by cells from the initial activation (E) and reactivation (F) are summarized from three independent experiments and donors (see Figure S3 for IL-2 and TNF-α quantification). (G and H) Quantification of CCR7^+^ CD27^+^ (G) and CCR7^–^ CD27^–^ (H) cells are summarized from three independent experiments. Error bars reflect SEM. *p < 0.05, **p < 0.01, paired two-tailed Student’s t test. ns, not significant.

We next examined how low glucose altered human T cell subsets’ ability to expand, adapt functionally, and differentiate. We sorted T_N_, T_CM_, and T_EM_ cells (Figure S2) and stimulated them with anti-CD3/CD28-coated beads in the presence of optimal glucose (35 mM) or low glucose (0.35 mM). As expected, all T cell subsets had substantially reduced growth in low glucose (Figure 2A). We next examined the ability of freshly sorted human T cell subsets to produce IFN-γ in the presence of optimal or low glucose after phorbol 12-myristate 13-acetate (PMA)/ionomycin stimulation. T_N_ and T_CM_ cells made less IFN-γ in low glucose, consistent with previous reports (Cham and Gajewski, 2005; Chang et al., 2013, 2015a; Ho et al., 2015). However, T_EM_ cells made equivalent levels of IFN-γ (Figures 2B and 2E), suggesting that the effector functions of T_EM_ cells are not compromised in poor nutrient environments. Interestingly interleukin-2 (IL-2) and tumor necrosis factor α (TNF-α) (Figures 2B, 2C, and S3) were not affected by glucose availability. Therefore, we expanded T cells from Figure 2B for 10–12 days and determined their ability to make IFNγ and IL-2 upon re-stimulation. We observed expanded T_N_ cells still have a profound defect in their ability to produce IFN-γ in low glucose, whereas both expanded T_CM_ and T_EM_ cells produced equivalent amounts of IFN-γ after being expanded in both optimal and low glucose (Figures 2C and 2F). The ability of expanded, but not freshly isolated, T_CM_ cells to make high levels of IFN-γ in the presence of optimal or low glucose is likely tied to their more differentiated phenotype after expansion (Figures 2D, 2G, and 2H). Furthermore, we found that while all cells upregulate CD25, the IL-2 receptor, following anti-CD3/CD28 bead activation in both optimal and low glucose, T_N_ and T_CM_ cells lost CD25 expression quickly following activation in low glucose, whereas T_EM_ cells were able to maintain heightened CD25 expression in limiting glucose (Figure S4), suggesting that they can maintain an activated phenotype in poor nutrient conditions. Cumulatively these data show that T_EM_ cells maintain function in low glucose, whereas T_N_ and T_CM_ cells make less IFN-γ and lose CD25 expression.

### Effector Memory T Cells Do Not Augment Oxidative Phosphorylation in Low Glucose

**Figure 3:**
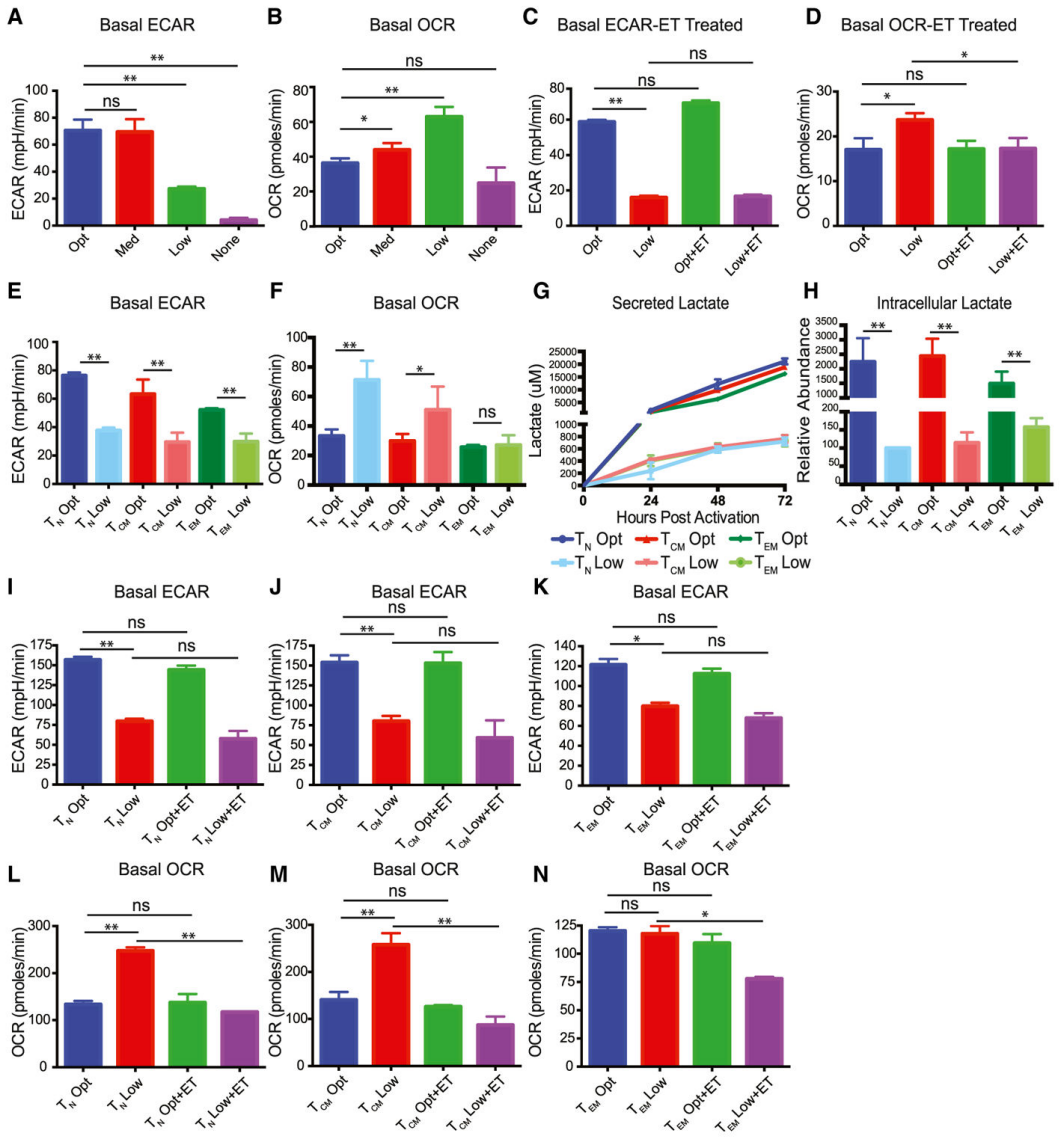
Effector Memory T Cells Are Unable to Augment Oxidative Phosphorylation in Low Glucose (A and B) Total CD4 T cells were activated with anti-CD3/CD28 coated beads in optimal (35 mM), medium (3.5 mM), low (0.35 mM), or no glucose (0 mM) for 48 hr, and basal extracellular acidification rate (ECAR) (A) and basal oxygen consumption rate (OCR) (B) were measured by XF Seahorse Analyzer. (C and D) Total CD4 T cells were activated with anti-CD3/CD28-coated beads in optimal and low glucose and pre-treated in the presence of etomoxir (ET) or vehicle (DMSO) for 48 hr before basal OCR (D) and ECAR (C) was measured by XF Seahorse Analyzer. (E and F) Basal OCR (E) and basal ECAR (F) of indicated T cell subsets were quantified. (G) Lactate was measured in the media of indicated T cell subsets after 24, 48, or 72 hr of culture using high-performance liquid chromatography (HPLC). (H) Relative intracellular abundances of lactate from sorted activated T cell subsets at 48 hr by LC-MS, normalized by cell number and cell volume. (I–N) Basal OCR rates of T_N_ (I), T_CM_ (J), and T_EM_ (K) and basal ECAR rates of T_N_ (L), T_CM_ (M), and T_EM_ (N) were quantified. Error bars reflect SEM. All data are representative of 4 independent experiments and donors. *p < 0.05, **p < 0.01, paired two-tailed Student’s t test or in case of multiple comparisons, one-way ANOVA followed by Tukey LSD; ns, not significant.

Past studies have linked T cell metabolism to IFN-γ production (Cham and Gajewski, 2005; Chang et al., 2013; Ho et al., 2015). To better understand the relationship between T cell metabolism and function, we performed metabolic assays on activated human T cells cultured in media with 35, 3.5, or 0.35 mM glucose or no glucose. In agreement with previous studies, we found that T cells cultured in lower amounts of glucose had reduced glycolysis as measured by extracellular acidification rate (ECAR) and compensated for this loss in glycolysis by upregulating oxidative phosphorylation measured by oxygen consumption rate (OCR; Figures 3A and 3B). Previous reports have demonstrated the importance of fatty acid oxidation in T cells to their ability to utilize and upregulate their OCR (O’Sullivan et al., 2014; van der Windt et al., 2012). We also found that OCR increases were dependent upon fatty acid oxidation as the increase in OCR in limiting glucose was blocked by etomoxir, a drug that blocks fatty acid intake into the mitochondria by inhibiting carnitine palmitoyltransferase (Figure 3D). However, etomoxir treatment did not significantly alter ECAR or intracellular or extracellular lactate production (Figure 3C, 3G, and 3H). T_N_ and T_CM_ cells behave in a similar fashion to total T cells by exhibiting reduced glycolysis and augmented OCR when cultured in low glucose (Figures 3E and 3F). Furthermore, etomoxir inhibited the OCR of all T cell subsets only in low glucose, without altering their glycolytic rate (Figures 3I–3N). These data show that effector memory T cells metabolically respond to low glucose distinctly from other T cell subsets by not augmenting oxidative metabolism.

### Effector Memory T Cells Contain Fewer Lipid Droplets than Other T Cell Subsets in Optimal Glucose

**Figure 4:**
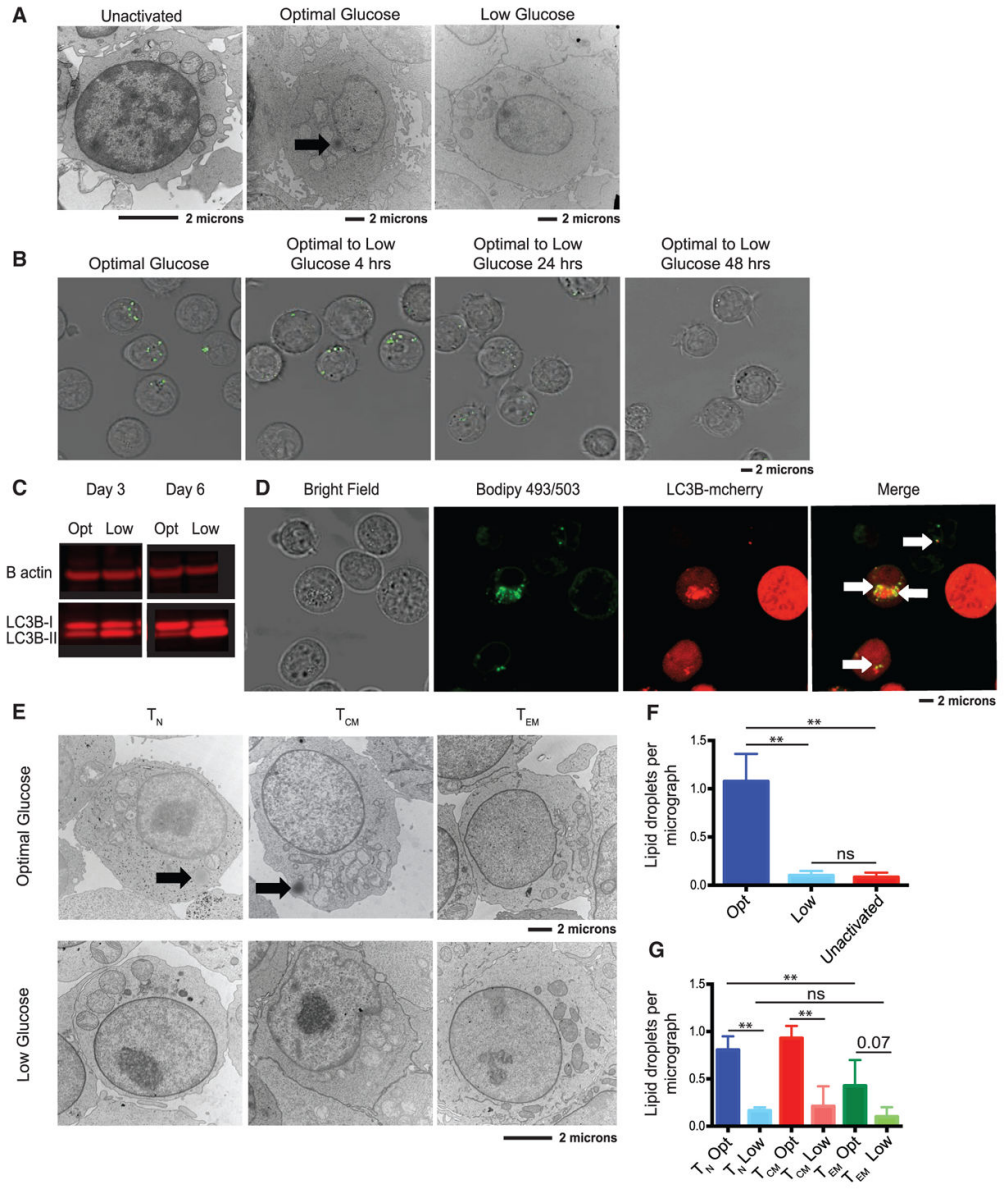
Effector Memory T Cells Contain Fewer Lipid Droplets than Other T Cell Subsets at Optimal Glucose (A) Total CD4 T cells were left unactivated or activated with anti-CD3/CD28-coated beads in optimal or low glucose. After 2 days of culture, T cells were examined using transmission electron microscopy. Arrow indicates presence of lipid droplets. Scale bars, 2 μm. (B) Total CD4 T cells were activated with anti-CD3/CD28-coated beads in optimal glucose for 48 hr and then transferred into medium with low glucose and cultured for up to an additional 48 hr. Bodipy 493/503 was used to stain the cells at the indicated time points after being transferred to low glucose for 0, 4, 24, or 48 hr. Fluorescence was visualized via confocal microscopy; 30–40 randomly selected cells per experiment were imaged (see Figure S5 for quantification). (C) Total CD4 T cells were activated with anti-CD3/CD28-coated beads for 3 or 6 days in optimal or low glucose. Cell lysates were probed for LC3B isoforms and β-actin (see Figure S5 for quantification). Data are representative of 3 independent experiments and donors. (D) T cells were stimulated with anti-CD3/CD28-coated beads and transduced with LC3B-mcherry. After 48 hr of activation, cells were stained with bodipy 493/503, and co-localization was visualized via confocal microscopy. 30–40 randomly selected cells per experiment were imaged. Data are representative of 3 independent experiments and donors. (E) Indicated subsets were stimulated with anti-CD3/CD28-coated beads in the presence of optimal or low glucose. After 2 days of culture, T cells were examined using transmission electron microscopy. Scale bar, 2 μm. (F and G) The number of lipid droplets per micrograph of total T cells (F) or indicated subsets (G) were quantified from 40 images per group per experiment in two independent experiments. Error bars reflect SEM. *p < 0.05, **p < 0.01, one-way ANOVA followed by Tukey LSD; ns, not significant.

Because T_N_ and T_CM_, but not T_EM_, cells could compensate for low glucose by augmenting oxidative phosphorylation in a fatty-acid-dependent manner, we wanted to examine the supply of lipids stored within the cell. Lipid droplets are dynamic organelles that play a key role in the regulation of lipid metabolism and serve as a ready to use source of lipids (Barneda and Christian, 2017). We first isolated total CD4 T cells and examined the number of lipid droplets in resting and activated cells in both optimal and low glucose. T cell activation was required for the formation of lipid droplets, as we did not observe lipid droplets in resting T cells. Moreover, we found that activated CD4 T cells grown in optimal glucose had dramatically more lipid droplets per cell than cells activated in low glucose (Figures 4A and 4F). We hypothesized that T cells expanded in low glucose could not store lipid droplets, because they were using lipids as fuel for oxidative phosphorylation. To determine whether lipids were being consumed in T cells when cultured in low glucose, we first activated T cells in optimal glucose for 48 hr and then placed them in low glucose for an additional 0, 4, 8, 24, or 48 hr. We further quantified lipid droplets by staining cells with the lipophilic dye bodipy 493/503, which is commonly used to mark neutral lipids (Singh et al., 2009). Significant decreases in bodipy florescence of stained lipid droplets were observed after placing cells in low glucose for more than 24 hr, indicating that T cells consume lipid droplets when placed in nutrient-poor conditions (Figures 4B and S5). After 6 days of culture, we observed significant number of autophagosomes (Figure S5). Under normal conditions, autophagic protein microtubule-associated protein 1 light chain-3B (LC3B) consists largely of its cytoplasmic form, LC3B-1. However, during autophagy, LC3B becomes conjugated to phosphatidylethanolamine, forming LC3B-II, and is recruited to the membrane of autophagosomes (He et al., 2003). We found that T cells cultured in optimal glucose maintained higher levels of LC3B-I than LC3B-II, whereas T cells cultured in low glucose had much more LC3B-II than LC3B-I (Figures 4C and S5), confirming that T cells were performing autophagy in response to low glucose.

Previous studies have implicated autophagy in fatty acid consumption (Liu and Czaja, 2013; Singh et al., 2009). We found that T cells were unable to consume their lipid droplets in low glucose when treated with an autophagy inhibitor, Lys05 (Figure S5). To further investigate whether autophagy was responsible for the breakdown of lipid droplets, we transduced T cells with a lentiviral vector expressing LC3B fused to mCherry, a pH-insensitive fluorescent reporter. We found that lipid droplets marked by bodipy 493/503 often co-localized with the autophagosomes (Figure 4D), suggesting that autophagy was responsible for lipid droplet breakdown and consumption. Furthermore, we found that inhibiting mTORC1 via rapamycin, a well-known method of inducing autophagy, increased T cell growth in low glucose but not in optimal glucose. We found that the enhanced growth by rapamycin was blocked when co-administering Lys05, while expansion was not significantly inhibited by Lys05 alone in low glucose (Figure S5). These data support the idea that promoting autophagy can increase T cell expansion in low glucose possibly by increasing lipid consumption. Next, we isolated T cell subsets to examine if T_EM_ cells may fail to upregulate oxidative phosphorylation because they cannot uptake lipids and store them as well as T_N_ or T_CM_ cells. We observed T_N_ and T_CM_ cells cultured in optimal glucose had significantly higher amounts of lipid droplets per cell per micrograph than T_EM_ cells (Figures 4E and 4G). These data demonstrate that T_EM_ cells acquire fewer lipid droplets in optimal glucose, suggesting that lipids may not be the preferred alternative fuel for T_EM_.

### Effector Memory T Cells Are Unable to Perform Reductive Glutaminolysis for Fatty Acid Synthesis When Cultured in Low Glucose

**Figure 5:**
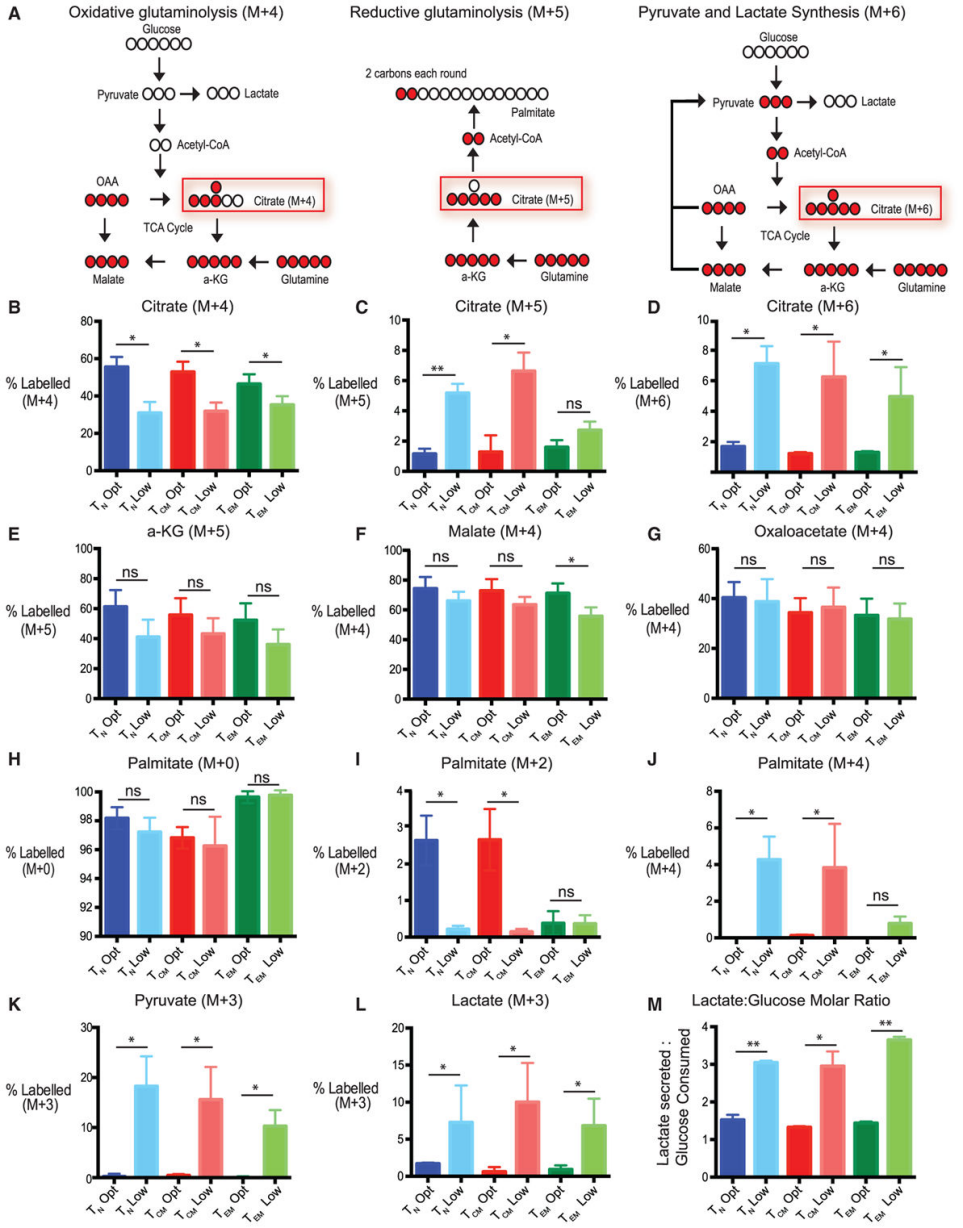
Effector Memory T Cells Cannot Perform Reductive Glutaminolysis for Fatty Acid Synthesis in Low Glucose (A) Model depicting how heavy glutamine is incorporated into citrate in an M+4, M+5, or M+6 manner and how each of those inform how glutamine is routed intracellularly. (B–D) Indicated T cell subsets were activated with anti-CD3/CD28-coated beads in optimal or low glucose supplemented with heavy glutamine for 48 hr. Percentage of M+4 (B), M+5 (C), or M+6 citrate (D) calculated from the total intracellular citrate pool of each subset by LC-MS is indicated. (E–G) Indicated T cell subsets were treated as in (B). Graphs show percentage of M+5 α-ketoglutarate (E), M+4 malate (F), and M+4 oxaloacetate (G) calculated from the total respective intracellular pools of each metabolite for each subset by LC-MS. (H–J) Indicated T cell subsets were treated as in (B). Percentages of M+0 (H), M+2 (I), and M+4 palmitate (J) were calculated from the total intracellular pool of palmitate for each subset by LC-MS. (K and L) Indicated T cell subsets were treated as in (B). Percentage of M+3 pyruvate (K) and M+3 lactate (L) from the total respective intracellular pool of each subset by LC-MS is indicated. (M) Ratios of lactate secreted and glucose consumed were calculated from moles of lactate secreted and moles of glucose consumed in supernatant of T cell subsets determined by HPLC 48 hr post-activation. Error bars reflect SEM. All data are representative of 3–4 independent experiments. See Figure S6 for overall relative metabolite abundances of each subset. *p < 0.05, **p < 0.01, paired two-tailed Student’s t test; ns, not significant.

Next, we wished to examine whether other salvage pathways were differentially regulated in T cell subsets in response to low glucose. An increased consumption of glutamine has been shown to be a salvage pathway to drive T cell metabolism in low glucose (Blagih et al., 2015). We quantified the rates of consumption/production of glucose, amino acids, and a few organic acids in the supernatant of activated T cells (Tables S3A and S3B). As expected, T cells primarily consumed glucose and glutamine. Additionally, we observed high consumption of serine, supporting recent findings that serine is essential for *de novo* nucleotide biosynthesis (Ma et al., 2017). T cells in low glucose regardless of subset, consumed fewer amino acids, likely due to overall reduced level of T cell activation. Interestingly glutamine was equally consumed by T cells at optimal or low glucose; thus, relative to other amino acids, glutamine was preferentially consumed by T cells cultured in low glucose. Surprisingly, tricarboxylic acid (TCA) cycle intermediates demonstrated different patterns of abundance, suggesting complex regulation of the TCA under nutrient stress (Figure S6). To examine how glutamine was utilized, we cultured T cells in the presence of labeled glutamine and examined how it was metabolized within the cell. The differential labeling of citrate can be used to interpret glutamine metabolic pathways (Figure 5A) (Fendt et al., 2013; Metallo et al., 2011). A citrate with 4 heavy carbons (marked mass +4 or M+4) suggested typical oxidative glutaminolysis, where glutamine is incorporated into the TCA cycle and then spread across other TCA cycle intermediates. We found that T cells cultured in low glucose had less labeled M+4 citrate, and this was observed in all T cell subsets examined (Figure 5B). To extend this finding, we examined other TCA intermediates and found equivalent levels of M+4 labeling, suggesting that oxidative glutaminolysis functions similarly in all T cell subsets in both optimal and low glucose (Figures 5E–5G).

A citrate with 5 heavy carbons suggests reductive glutaminolysis where glutamine is being routed into fatty acid synthesis (Figure 5A). We found that T_N_ and T_CM_ cells had higher relative amounts of citrate M+5 when cultured in low glucose, suggesting that some of the glutamine imported into T cells was being converted to fatty acids (Figure 5C). T_EM_ cells, on the other hand, did not to appear to upregulate fatty acid synthesis in the presence of low glucose, since the relative percentage M+5 was the same regardless of the T cells being cultured in optimal or low glucose. To confirm this, we examined both M+2 and M+4 palmitate, which would directly show glutamine being converted to fatty acids. Interestingly, we observed that T_N_ and T_CM_ cells expanded in optimal glucose had higher levels of M+2 palmitate than their respective groups grown in low glucose but higher M+4 palmitate (Figures 5H–5J). In contrast, T_EM_ cells have low levels of M+2 palmitate when cultured in both optimal and low glucose, suggesting that very little glutamine gets converted to fatty acids in T_EM_ regardless of the glucose levels present. These data indicate that reductive glutaminolysis is occurring at a higher rate in T_N_ and T_CM_ cells cultured in low glucose. Only small amounts of M+4 palmitate was detected in T_EM_ cells, further confirming very little glutamine is converted to fatty acids in T_EM_ regardless of the glucose levels present.

A citrate with 6 heavy carbons suggests that some fraction of glutamine is being converted into pyruvate and this pyruvate is then being used to generate citrate (Figure 5A). We found that all T cell subsets expanded in low glucose had higher levels of citrate M+6, indicating that the differential use of glutamine by T_EM_ cells is confined to reductive glutaminolysis (Figure 5D). Interestingly, all T cells subsets redirect glutamine into production of pyruvate by having increased M+3 labeling of pyruvate, making an estimated 20% of the intracellular pool in low glucose (Figure 5K). Additionally, ~10% of the intracellular pool of lactate is made from glutamine, suggesting that this glutamine-derived pyruvate is converted into lactate (Figure 5L). We found that all subsets when cultured in optimal glucose secreted ~1.5 mol of lactate per mole of glucose consumed, consistent with previous reports showing that most of the glucose utilized is converted into lactate (Frauwirth et al., 2002). However, when T cells were cultured in low glucose, we found that lactate/glucose ratios exceeded 2, suggesting that a carbon source other than glucose was being used to produce lactate (Figure 5M). Together, T cells appear to be adept at using glutamine to fulfill a diverse array of metabolic needs when glucose is limiting with the notable exception that T_EM_ cells are unable to use glutamine to produce fatty acids.

### Effector Memory T Cells Are Less Reliant on Fatty Acid Metabolism for Survival and Expansion at Low Glucose

**Figure 6:**
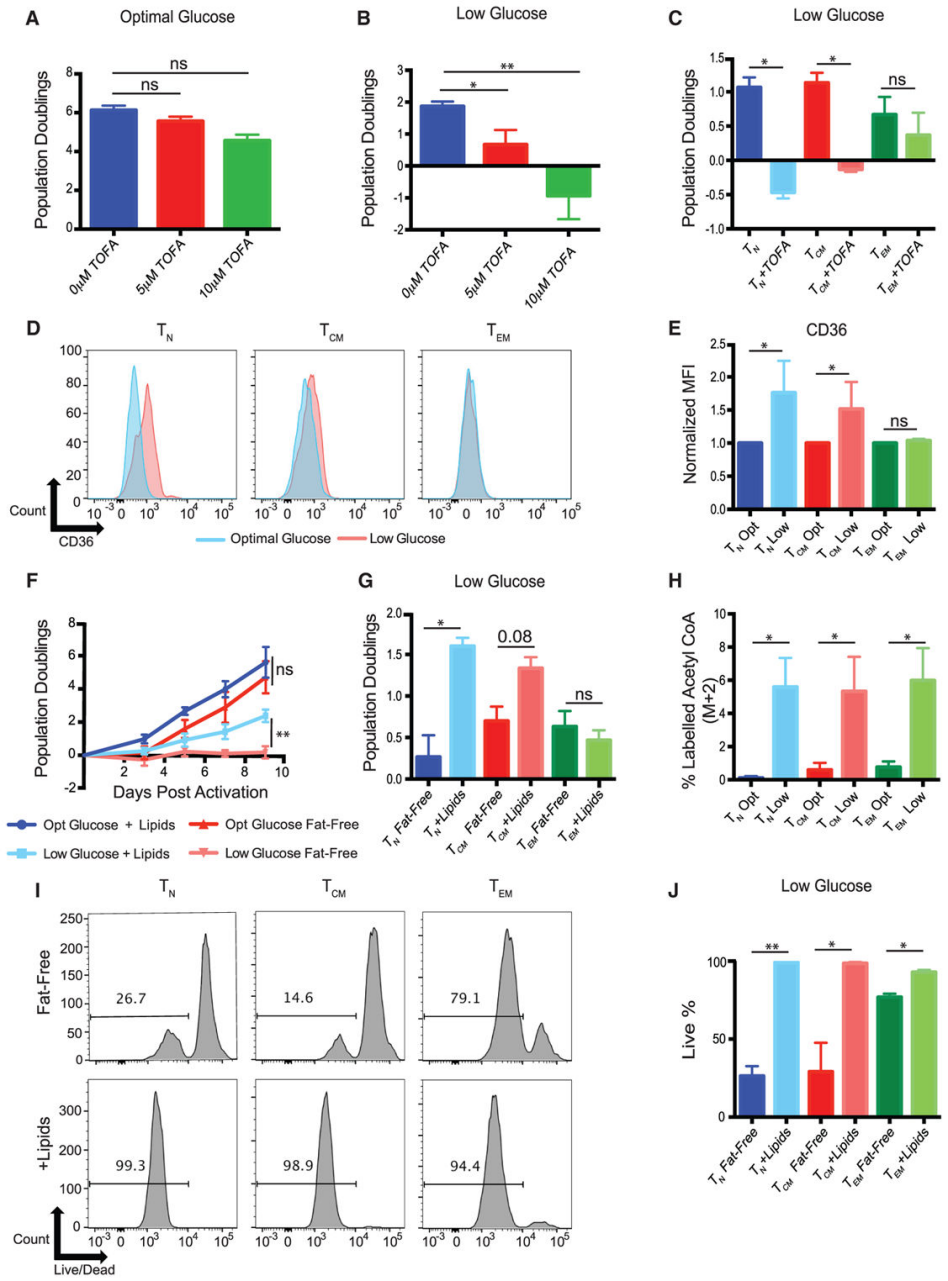
Effector Memory T Cells Are Less Reliant on Fatty Acid Metabolism for Survival and Expansion in Low Glucose (A and B) Total CD4 T cells were activated with anti-CD3/CD28-coated beads in optimal (A) or low glucose (B) in the presence of TOFA or with vehicle (DMSO) for 5 days. Total cell expansion is recorded on day 5 post-activation. (C) Indicated T cell subsets were activated with anti-CD3/CD28-coated beads in low glucose in the presence of TOFA or vehicle DMSO for 5 days. Cell expansion is recorded on day 5 post-activation. (D) Indicated T cell subsets were activated with anti-CD3/CD28 coated beads in optimal or low glucose and CD36 expression was measured at 48 hr post-activation. (E) Quantification of (D) from three independent experiments and donors. (F) Total CD4 T cells were activated with anti-CD3/CD28 beads in optimal or low glucose in media without any exogenous lipids (fat-free) or supplemented with exogenous lipids for 9 days. Cell expansion was monitored by Coulter counter on the days indicated. (G) Indicated T cell subsets were activated with anti-CD3/CD28-coated beads in low-glucose, fat-free medium with or without supplementation of exogenous lipids for 5 days. Cell expansion was recorded on day 5 post-activation. (H) Indicated subsets were treated with heavy palmitate for 24 hr. Percentages of M+2 acetyl CoA were calculated from the total acetyl-CoA pool by LC-MS. (I) Indicated T cell subsets were activated with anti-CD3/CD28-coated beads in fat-free medium in low glucose with or without exogenous lipids. Live cells were identified with Live/Dead Aqua by flow cytometry on day 5 post-activation. (J) Quantification of live cells from (H). Error bars reflect SEM. All data are representative of 3 independent experiments. *p < 0.05, **p < 0.01, paired two-tailed Student’s t test or in case of multiple comparisons, one-way ANOVA followed by Tukey LSD; ns, not significant.

With evidence of fatty acid droplets being consumed in low glucose and data demonstrating that glutamine was being redirected into fatty acid synthesis by T_N_ and T_CM_ cells, we sought to explore the importance of fatty acid metabolism for the survival and expansion of T cells in low glucose. We treated activated T cells in optimal or low glucose with 5-(tetradecyloxy)-2-furoic acid (TOFA), an inhibitor of fatty acid synthesis, or vehicle (DMSO). We saw little to no effect of TOFA on T cell expansion in optimal glucose and significant inhibition of T cell expansion in low glucose (Figures 6A and 6B). We further examined how TOFA affected the expansion of T cell subsets grown in low glucose. T_N_ and T_CM_ cells behaved in a similar manner to total T cells and suffered severe proliferative defects when cultured in low glucose (Figure 6C). In contrast, expansion of T_EM_ cells was only marginally affected by TOFA in both optimal and low glucose. However, T cells can also uptake fatty acids from exogenous sources (O’Sullivan et al., 2014). We quantified surface expression of CD36, a scavenger receptor that is thought to be important for uptake of long-chain fatty acids (Glatz and Luiken, 2017; Schwenk et al., 2010). We observed that T_N_ and T_CM_ cells could upregulate CD36 when grown in low glucose, while T_EM_ cells did not (Figures 6D and 6E). Together, these data suggest that T_EM_ cells are not as reliant as T_N_ or T_CM_ cells on fatty acid synthesis or uptake of exogenous fatty acids to expand in limiting glucose.

To further characterize which T cell subsets, if any, are dependent on exogenous fatty acids, we prepared fully defined, serum-free media that lacked all species of lipids (fat-free). Despite an early defect in proliferation, we found that total T cells in optimal glucose in the absence of lipids expanded nearly as well as T cells in the presence of lipids (Figure 6F). However, in low glucose, the lack of fatty acids severely impaired T cell expansion. This pattern remained consistent in T_N_ and T_CM_ cells, and their expansion was severely inhibited in fat-free media when grown in low glucose (Figure 6G). Surprisingly, T_EM_ cells grew equally well in the presence and absence of lipids in low glucose. Next, we wanted to quantify how much different T cell subsets incorporated exogenous lipids into the TCA cycle. In agreement with our data in Figure 2, we found that all subsets equally increased their incorporation of exogenous fats into acetyl CoA (Figure 6H). We examined the viability of T cells and observed that most of the T_N_ and T_CM_ cells were dead in low glucose, fat-free media, whereas T_EM_ cells had near-equivalent viability in the presence and absence of lipids (Figures 6I and 6J). Cumulatively, these results show that T_EM_ cells are not as reliant as T_N_ or T_CM_ cells on lipid metabolism to expand or survive in low glucose.

### Impairment of Fatty Acid Synthesis in Naive T Cells Augments IFN-γ Expression

**Figure 7:**
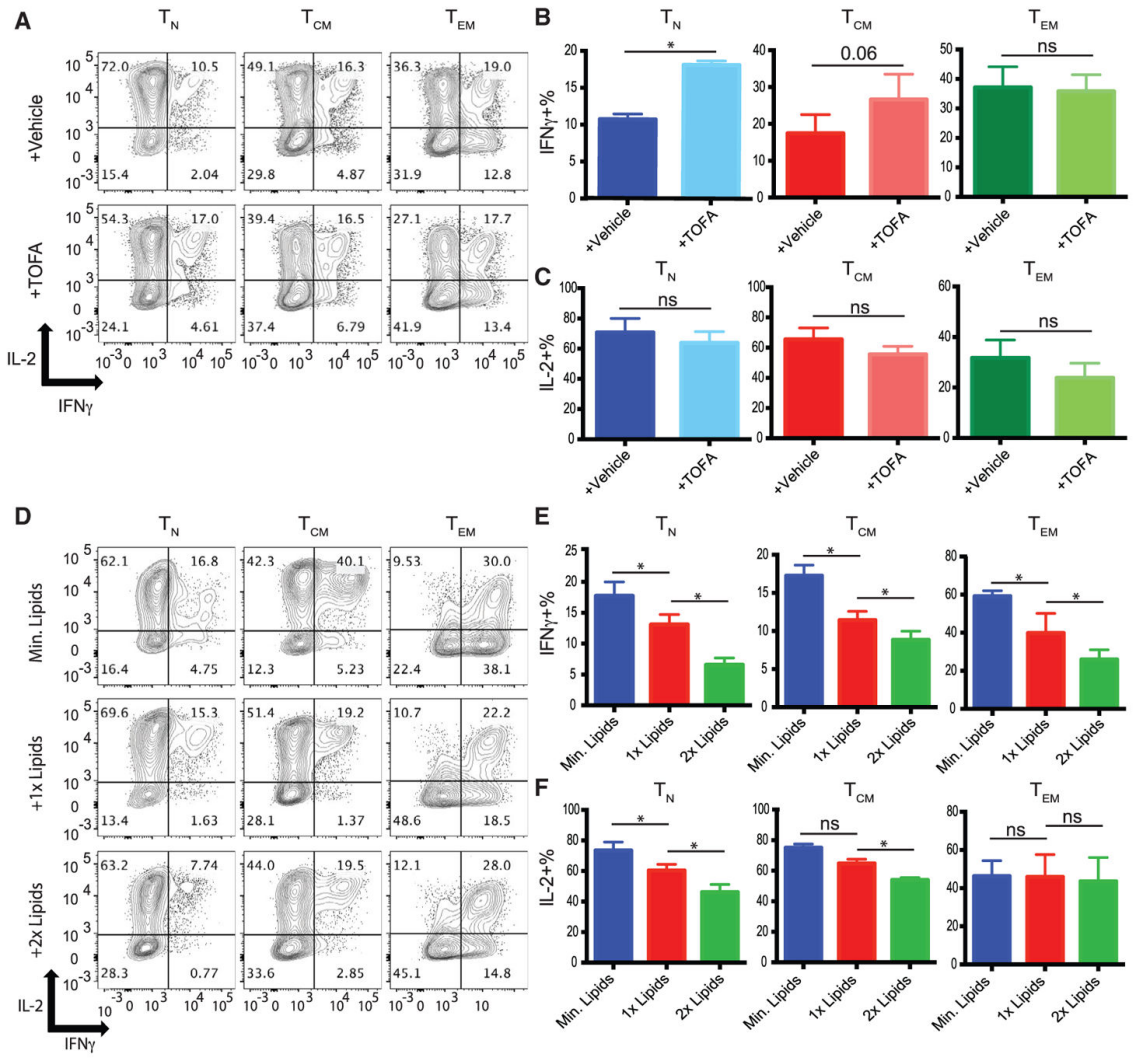
Reliance on Fatty Acid Metabolism in Low Glucose Inhibits IFN-γ Production (A) Indicated T cell subsets were activated with anti-CD3/CD28-coated beads in low glucose in the presence of vehicle (DMSO) or low-dose TOFA for 5 days before IFN-γ and IL-2 production was measured after PMA/ionomycin treatment. For optimal glucose data, see Figure S7. (B and C) Quantification of IFN-γ (B) and IL-2 (C) production from 4 independent experiments. (D) Indicated T cell subsets were activated with anti-CD3/CD28-coated beads in low glucose in the presence of minimal, 1×, or 2×exogenous lipid concentrate in fat-free medium for 5 days post-activation before IFN-γ production was measured following PMA/ionomycin treatment. (E and F) Quantification of IFN-γ (E) and IL-2 (F) production from 3 independent experiments. Error bars reflect SEM. *p < 0.05, **p < 0.01, paired two-tailed Student’s t test; ns, not significant.

Our data demonstrate a strong correlation between a T cell subset’s ability to utilize fatty acids and subsequent ability to produce IFN-γ in limiting glucose. To determine whether there is a relationship between IFN-γ expression and fatty acid metabolism, we first asked whether a reduction in fatty acid synthesis would augment IFN-γ production. To do this, we used a dose of TOFA (5 μM) that permits T_N_ cells to survive in low glucose and then examined their ability to make IFNγ. Blocking fatty acid synthesis in T_N_ cells significantly increased their ability to make IFN-γ (Figures 7A and 7B). This did not, however, impact T_EM_ cells ability to produce IFN-γ, further confirming the notion that T_EM_ cells are not actively synthesizing fatty acids and thus are insensitive to TOFA. Interestingly, blocking fatty acid synthesis did not impact the production of other cytokines such as IL-2 in any of the subsets (Figure 7C), suggesting that IFN-γ is specifically targeted by fatty acid metabolism. Furthermore, TOFA did not alter IFN-γ or IL-2 production in T_N_ cells or any other subset in optimal glucose (Figure S7). This is consistent with our mass spectrometry data, suggesting that T cells activated in optimal glucose do not utilize fatty acid synthesis to a significant extent.

Next, we sought to determine whether increasing the concentration of exogenous lipids in the media would cause decreased production of IFN-γ by T cells. To do this, we added a defined minimal lipid concentration that permitted T cell expansion in low glucose to our lipid-free media. To investigate how individual subsets reacted to the presence of exogenous lipids, we added increasing doses of exogenous lipids to sorted subsets and examined IFN-γ and IL-2 production. We found that in minimal lipids, IFN-γ production by all subsets was higher than that of cells in normal amounts of lipids (1×) in low glucose (Figures 7D and 7E). Furthermore, we found that as we increased lipid concentration, IFN-γ production further decreased in all subsets. Moreover, the addition of exogenous lipids caused decreases in not only IFN-γ but also IL-2 in T_N_ and T_CM_ cells, but not T_EM_ cells (Figures 7C–7E), further highlighting T_EM_ cells’ relative resistance to modulate their effector functions due to fatty metabolism. Together, these data demonstrate that lipid metabolism regulates IFN-γ production.

## DISCUSSION

Over the past few years, it has become increasingly clear that T cell metabolism plays a crucial role in driving T cell differentiation and function (Pollizzi and Powell, 2014). Our studies uncovered that T cell subsets respond distinctly to the same metabolic stress. In response to low glucose, T_N_ and T_CM_ cells increase oxidative phosphorylation, rely on fatty acid metabolism, increase autophagy, and redirect glutamine into pathways that produce both fatty acids and pyruvate. We show that unlike T_N_ or T_CM_ cells, T_EM_ cells do not rely on fatty acid synthesis or increase oxidative phosphorylation in low glucose. We further demonstrate that by being less reliant on fatty acid pathways T_EM_ cells can maintain functionality during nutrient stress.

Our studies suggest that the relative ratio of glycolysis to fatty acid metabolism within a single effector T cell determines its functional capabilities. By limiting fatty acid metabolism in T_N_ cells, we could significantly augment their IFN-γ production. Conversely, by culturing T_EM_ cells in high levels of lipids, we selectively decreased their ability to make IFN-γ. Thus, our studies show a strong link between active fatty acid metabolism and the ability to make IFN-γ, adding to a number of mechanisms a T cell employs to regulate IFN-γ production (Chang et al., 2013; Peng et al., 2016; Siska and Rathmell, 2016). There are many challenges associated with translating *in vitro* findings on T cell metabolism to *in vivo* models (C.E. and J.L.R., unpublished data). Simple adoptive transfer studies favor less differentiated T cell due to their ability to expand and differentiate into effector memory T cells (Gattinoni et al., 2011). Models in which the metabolic milieu and the number infiltrating T lymphocytes can be carefully controlled and monitored will be necessary to confirm our findings *in vivo*. To date, the best *in vivo* data supporting our *in vitro* studies come from studies that correlate the number of T_EM_ cells within the tumor microenvironment with patient survival (Farber et al., 2014; Pagès et al., 2005; Thome et al., 2014).

Immune cells are one of the few groups of cells in the body that must travel to a wide spectrum of environments. These environments have a diverse array of metabolic requirements, and immune cells must be able to function in all of them. Our work and that of others demonstrate that T cells can downregulate activation, proliferation, and transcription to lower energy consumption while relying on salvage pathways to utilize diverse fuel sources (Blagih et al., 2015). Previous work has demonstrated not only that glucose is essential for generating energy quickly through glycolysis but also that glycolytic intermediates are necessary for effective T cell activation and proliferation (Ho et al., 2015). Thus, these salvage pathways do not simply need to meet the energy demands for the cell when traditional nutrients are scarce; rather, they need to supply the building blocks to generate proteins, fatty acids, and nucleic acids required for T cell expansion and differentiation (Pearce and Pearce, 2013). Our data that glutamine can be shunted into all metabolic pathways examined in glucose-limiting conditions, driving increased oxidative phosphorylation and increased biosynthetic production of fatty acids and pyruvate, demonstrates how different carbon sources could be utilized in nutrient-poor conditions. Increased oxidative phosphorylation in limiting glucose likely amplified the need for fatty acid oxidation and exogenous fatty. The incredible flexibility of T cells to manufacture the building blocks of many synthetic pathways underlies their ability to function throughout the body. Our studies highlight how the salvage pathways that T cells choose to alter their eventual functionality. Understanding the metabolic demands of T cell subsets and how to modulate traditional and salvage metabolic pathways may yield more effective cellular therapies for cancer and other diseases.

## EXPERIMENTAL PROCEDURES

### Study Design

The purpose of this study was to characterize human T cell subsets and identify metabolic and functional outcomes when grown in sufficient and deficient conditions. The number of replicates per experiment is indicated in the figure legends and performed in a controlled and non-blinded manner.

### Immune Cell Purification and Sorting

De-identified human CD4 T cells were obtained from the Human Immunology Core at the University of Pennsylvania under an institutional review board (IRB)-approved protocol and stained using antibodies for CD4 (BD PharMingen, 562424), CD45RA (BD PharMingen, 337167), CD25 (BD PharMingen, 557138), CCR7 (BioLegend, 353218), and CD27 (BioLegend, 353218). T_N_ (CD45RA^+^CCR7^+^CD27^+^CD25^−^), T_CM_ (CD45RA^−^CCR7^+^CD27^+^CD25^−^), and T_EM_ cells (CD45RA^−^CCR7^−^CD27^−^CD25^−^) were sorted to high purity using a BD FACS AriaII.

### Cell Culture and Activation

Sorted CD4 T cells were washed twice in PBS and then placed in IB2H serum-free medium containing optimal (35 mM), medium (3.5 mM), or low (0.35 mM) glucose concentrations. The medium was supplemented with 1× (500 μL), 2× (1,000 μL), or 3× (1,500 μL), when indicated, of a chemically defined lipid mixture (ThermoFisher Scientific, 11905031; individual lipid concentrations available online) and 8 mM L-glutamine. Cells were then activated at 1 million cells/mL using Dynabeads Human T-Expander CD3/CD28 (ThermoFisher Scientific, 11131D) at a concentration of 3 beads per cell. Additional volumes of medium were added on day 3 and every day after so that each culture was at 0.5 million cells/mL after feeding. Cells were treated with TOFA (5 or 10 μM; Sigma Aldrich, T6575), or etomoxir (200 or 400 μM; Sigma Aldrich, E1905). cDNA encoding LC3B was synthesized (IDT) and transferred into pTRPE, a lentiviral transfer vector (Leibman et al., 2017). Lentiviral supernatants and T cell transduction were generated and performed as previously described (Parry et al., 2003)

### Intracellular Cytokine Staining

Sorted cells were treated with 1 μg PMA (Sigma Aldrich, P1585) per milliliter of media, 3 μg ionomycin (Sigma Aldrich, 407950) per milliliter of media, and GolgiStop (BD PharMingen 554724) for 6 hr at 37°C immediately following sorting or 9–11 days post-activation after cells had rested and stopped dividing. Cells were stained with Live/Dead Aqua (ThermoFisher Scientific, L34957) according to manufacturer’s instructions. Cells were washed with 1× PBS and fixed using Fixation Medium A (ThermoFisher Scientific, GAS001S100) for 15 min at room temperature. Cells were then washed again with 1× PBS. Cells were then permeabilized using Permeabilization Medium B (ThermoFisher Scientific, GAS002S100) and stained with antibodies for IFN-γ (eBioSciences, 45-7319-42), IL-2 (BD PharMingen, 554567), and TNF-α (BD PharMingen 557647) for 15 min at room temperature. Cells were then washed 1× with PBS and analyzed using the BD LSR II flow cytometer.

### Western Blotting

Cells were lysed with 1× RIPA Buffer (Cell Signaling Technology, 9806) and 1 mM PMSF (Cell Signaling Technology, 8553S) according to manufacturer’s instructions. Proteins were resolved by SDS-PAGE and transferred to nitrocellulose. Blots were probed with anti-LC3B (Cell Signaling Technology, 3868) and anti-β-actin (Cell Signaling Technology, 4970). Protein was visualized using Odyssey CLx LI-COR instrument.

### Confocal Microscopy

To quantify neutral lipid droplets, cells were stained with 500 n/mL bodipy 493/503 (ThermoFisher Scientific, D3922) in serum free medium at 37°C for 30 min. Cells were then washed two times with PBS and placed in their respective media. Cells were live imaged at 37°C using a stage heater on the Leica TCS SP8 confocal microscope.

### Metabolite Extraction, Derivatization, and LC-MS Measurements

Cells were activated with anti-CD3/CD28 beads in media supplemented with 8 mM [U-^13^C]-glutamine (Cambridge Isotope Laboratories, CNLM-1275-H-PK) for 48 hr when indicated. The isolation and liquid chromatography- mass spectrometry (LC-MS) measurements of organic acids were performed as described previously with slight modification (Angelin et al., 2017; Guo et al., 2016). Briefly, cells were washed twice with PBS before extracting using 750 μL ice-cold methanol/water (4/1 v/v). For metabolites quantification, samples were spiked with internal standards (250 ng [^13^C_4_]-succinate, 250 ng [^13^C_6_]- citrate, 250 ng [^13^C_3_]-pyruvate, 1 μg [^13^C_3_]-lactate, 25 ng [^13^C_4_, ^15^N]-aspartate, 1 μg [^13^C_5_, ^15^N]-glutamate and 250 ng [^13^C_6_]-glucose 6-phosphate). Samples were pulse-sonicated for 30 s with a probe tip sonicator and centrifuged at 16,000 × *g* for 10 min. The supernatant was transferred to a new tube before evaporation to dryness under nitrogen. For quantifying pyruvate, α-ketoglutarate and oxaloacetate, 1 mg phenylhydrazine was included in the 750 μL methanol/water (4/1 v/v) for metabolite extraction. Samples were suspended in 50 μL before LC-MS analysis using an Agilent 1200 series high-performance liquid chromatography system coupled to an Agilent 6460 triple quadrupole mass spectrometer equipped with an electrospray ionization source. Analytes were separated by reversed-phase ion-pairing chromatography utilizing a Xselect HSS C18 column (150 × 2.1 mm, 3.5 μm, 100 Å; Waters). For samples that needed to be analyzed for the isotopic distribution of acetyl-CoA, cells were extracted using methanol/water as described above except that the samples were re-suspended in 50 μL of water with 5% 5-sulfosalicylic acid before LC-MS analysis. The quantification of acetyl-CoA was performed as described previously (Snyder et al., 2015), with slight modification. Briefly, cells were quenched with 750 μL of ice-cold 10% trichloroacetic acid (TCA) in water spiked with yeast extract labeled with [^13^C_3_^15^N_1_]-pantothenate. Samples were pulse-sonicated for 30 s with a probe tip sonicator and centrifuged at 16,000 × *g* for 10 min. The supernatants were loaded to Oasis HLB 1 cc (30 mg) SPE columns (Waters) conditioned with 1 mL methanol. After 1 mL wash with water, the samples were eluted with 1 mL of 25 mM ammonium acetate in methanol and dried under nitrogen. The samples were re-suspended in 50 μL water with 5% 5-sulfosalicylic acid. The acetyl-CoA was analyzed by an Ultimate 3000 autosampler coupled to a Thermo Q Exactive HF Hydro Quadrapole-Orbitrap mass spectrometer as previously described (Frey et al., 2016). The same LC conditions and column were used as described for the organic acid analysis.

### Transmission Electron Microscopy

Tissues for electron microscopic examination were fixed with 2.5% glutaraldehyde, 2.0% paraformaldehyde in 0.1 M sodium cacodylate buffer, pH7.4, overnight at 4°C. After subsequent buffer washes, the samples were post-fixed in 2.0% osmium tetroxide for 1 hr at room temperature and then washed again in buffer followed by water. After dehydration through a graded ethanol series, the tissue was infiltrated and embedded in EMbed-812 (Electron Microscopy Sciences, Fort Washington, PA). Thin sections were stained with uranyl acetate and lead citrate and examined with a JEOL 1010 electron microscope fitted with a Hamamatsu digital camera and AMT Advantage image capture software. To quantify lipid droplets, 30–40 cells per sample group were examined in two separate experiments, and lipid droplets were counted by eye.

### Seahorse XF Assay

OCR and ECAR were measured using a 96-well XF extracellular flux analyzer (Seahorse Bioscience). 250k cells per well were activated for 48 hr in optimal or low-glucose IB2H medium, washed with PBS, and transferred to warm, optimal, or low-glucose supplemented XF Seahorse medium.

### Statistics

Statistical analysis was performed using a 2-tailed Student’s t test or Mann-Whitney test after the analysis of distribution of variables. In the case of multiple comparisons, one-way ANOVA followed by Tukey least significant difference (LSD) was performed. Significance was determined at p < 0.05, and error bars indicate mean ± SEM. All calculations were made using GraphPad Prism 5 software (GraphPad Software) or Microsoft Excel.

## Supplementary Material

1

2

## Highlights

- T_EM_ cells do not inhibit IFN-γ production in a glucose-dependent manner
- T_EM_ cells are unable to use reductive glutaminolysis in low glucose
- Unlike T_N_ or T_CM_ cells, T_EM_ cells do not rely on fatty acid synthesis in limiting glucose
- Fatty acid metabolism inhibits IFN-γ production
