# Supplementary material for: Differential Reliance on Lipid Metabolism as a Salvage Pathway Underlies Functional Differences of T Cell Subsets in Poor Nutrient Environments
Source: Cell Rep. Author manuscript; Available in PMC 2018 May 1. (PMC5929999; doi:10.1016/j.celrep.2018.03.084)
Supplement: 1 [file NIHMS962084-supplement-1.pdf]

Cell Reports, Volume *23*

Supplemental Information

Differential Reliance on Lipid Metabolism as a Salvage Pathway Underlies Functional Differences of T Cell Subsets in Poor Nutrient Environments

Christopher Ecker, Lili Guo, Stefana Voicu, Luis Gil-de-Gómez, Andrew Medvec, Luis Cortina, Jackie Pajda, Melanie Andolina, Maria Torres-Castillo, Jennifer L. Donato, Sarya Mansour, Evan R. Zynda, Pei-Yi Lin, Angel Varela-Rohena, Ian A. Blair, and James L. Riley

A.

**Prototype Media**	**Base 1 Lean media with balanced AA and vitamins**	**Base 2 Non-essential AA, vitamins, trace elements and proteins**	**Base 3 Concentrated AA, vitamins, trace elements, metal ions, antioxidants, polyamines and lipids**
**1**	1.00	0.00	0.00
**2**	0.50	0.00	0.50
**3**	0.50	0.50	0.00
**4**	0.00	0.50	0.50
**5**	0.33	0.33	0.33
**6**	0.00	1.00	0.00
**7**	0.00	0	1
**8**	0.66	0.17	0.17
**9**	0.17	0.17	0.66
**10**	0.17	0.67	0.17

B.

X-VIVO™ 15 Media + 5% Serum	**Day 0 (% Remaining)**	**Day 3 (% Remaining)**	**Day 3 Post-Feed (% Remaining)**	**Day 6 (% Remaining)**
**L-Arginine**	100	62	91	52
**L-Glutamine**	100	58	91	28
**L-Leucine**	100	69	93	64
**L-Methionine**	100	62	91	52
**L-Serine**	100	7	78	nq
**L-Tryptophan**	100	26	91	nq
**Ethanolamine HCl**	100	nq	70	nq

C.

Prototype Media	**Day 0 (% Remaining)**	**Day 3 (% Remaining)**	**Day 3 Post-Feed (% Remaining)**	**Day 6 (% Remaining)**
**L-Arginine**	100	78	95	76
**L-Glutamine**	100	66	94	62
**L-Leucine**	100	67	91	58
**L-Methionine**	100	60	91	58
**L-Serine**	100	nq	83	14
**L-Tryptophan**	100	nq	92	nq
**Ethanolamine HCl**	100	48	96	40

Table S1, related to Figure 1: Creation of prototype media and spent media analysis for development of 1B2H medium.

A. Proportions of SFM1, SFM2, and SFM3 used to create ten media variants for 1B2H media creation are indicated. These ten media variants were then used in **Figure S1**. **B.** Total T cells were activated with anti-CD3/CD28 coated beads in XVIVO^{TM}-15 media supplemented with 5% human serum. Relative amounts of amino acids were analyzed at indicated time points by HPLC. Cells were counted on Day 3, and every other day afterward so that new media could be added to keep the concentration of cells at 500k cells/mL. nq = below limit of detection, not quantifiable. **C.** Total T cells were activated with anti-CD3/CD28 coated beads in 1B2H media. Relative amounts of amino acids were analyzed at indicated time points by HPLC. Cells were counted on Day 3, and every other day afterward so that new media could be added to keep the concentration of cells at 500k cells/mL. nq = below limit of detection, not quantifiable.

Glucose Concentration (mM)	Day 0 (% Remaining)	Day 3 (% Remaining)	Day 5 (% Remaining)	Day 7 (% Remaining)	Day 9 (% Remaining)
**35.0**	100	82.72727	68.78788	69.39394	72.72727
**24.5**	100	74.65619	55.59921	54.6169	38.25137
**17.5**	100	67.75956	36.61202	38.79781	29.46955
**10.5**	100	49.76744	1.395349	nq	nq
**7.0**	100	31.46853	nq	nq	nq
**3.5**	100	9.859155	nq	nq	nq
**0**	nq	nq	nq	nq	nq

Table S2, related to Figure 1: Determination of the optimal glucose concentration in 1B2H media.

Total T cells were activated with anti-CD3/CD28 coated beads in 1B2H media supplemented with the indicated amounts of glucose. Relative amounts of glucose remaining in the media were determined at the indicated time points by HPLC. Cells were counted on Day 3, and every other day afterward so that new media could be added to keep the concentration of cells at 500k cells/mL. nq= below limit of detection, not quantifiable.

A.

Metabolites consumed	T_N Optimal (mg/L)	T_N Low (mg/L)	T_{CM} Optimal (mg/L)	T_{CM} Low (mg/L)	T_{EM} Optimal (mg/L)	T_{EM} Low (mg/L)
**Glucose**	10785.74+/-1326.96	237.85+/-64.74	9257.99+/-1177.92	237.89+/-64.75	6333.86+/-607.04	78.28+/-21.31
**L-Glutamine**	288.31+/-17.49	278.38+/-13.77	270.38+/-9.02	264.85+/-7.86	260.23+/-13.89	272.65+/-17.42
**L-Serine**	26.45+/-3.59	14.70+/-4.58	24.15+/-1.79	11.77+/-1.71	15.74+/-0.60	9.98+/-1.19
**L-Lysine HCl**	16.14+/-1.42	6.25+/-1.80	17.61+/-1.72	4.93+/-1.62	12.75+/-1.53	5.91+/-1.71
**L-Arginine**	13.96+/-4.03	6.98+/-2.02	12.64+/-0.59	5.23+/-1.51	9.54+/-1.14	6.81+/-1.97
**L-Isoleucine**	9.60+/-2.77	5.64+/-1.63	9.95+/-0.78	4.71+/-2.33	7.85+/-0.89	5.31+/-1.53
**L-Leucine**	8.23+/-3.20	6.20+/-1.79	14.08+/-3.96	5.53+/-1.60	10.23+/-2.96	5.97+/-1.72
**L-Valine**	8.28+/-2.36	3.92+/-1.14	9.09+/-2.59	3.28+/-0.95	6.71+/-1.92	3.79+/-1.10
**L-Tyrosine**	5.28+/-1.52	2.29+/-0.66	5.51+/-1.52	1.54+/-0.44	4.04+/-1.15	2.04+/-0.59
**L-Methionine**	4.07+/-0.94	3.35+/-0.97	5.46+/-0.17	3.14+/-0.91	3.94+/-0.72	3.08+/-0.11
**L-Tryptophan**	3.76+/-1.08	2.87+/-0.83	3.32+/-0.91	2.33+/-0.67	2.70+/-0.74	2.94+/-0.85
**L-Phenylalanine**	3.24+/-1.91	3.06+/1.22	6.92+/-1.91	2.54+/-1.12	4.90+/-1.35	3.12+/-1.36

B.

Metabolites Produced	T_N Optimal (mg/L)	T_N Low (mg/L)	T_{CM} Optimal (mg/L)	T_{CM} Low (mg/L)	T_{EM} Optimal (mg/L)	T_{EM} Low (mg/L)
**Lactate**	12271.47+/-1471.81	587.28+/-41.94	9826.15+/-684.33	629.17+/-39.20	6373.29+/-377.56	616.05+/-61.78
**Ammonia**	25.43+/-6.14	34.52+/-7.57	24.14+/-6.46	33.08+/-9.39	22.61+/-6.37	28.77+/-7.13
**L-Glutamic Acid**	11.83+/-2.66	11.44+/-2.92	6.71+/-0.90	6.96+/-1.88	7.40+/-1.18	2.52+/-0.82
**L-Alanine**	1.17+/-0.51	8.43+/-1.56	1.84+/-1.01	8.55+/-2.31	1.50+/-0.42	3.65+/-1.37
**L-Aspartic Acid**	nq	4.51+-/1.49	nq	2.81+/-1.54	nq	2.51+/-1.96

Table S3, related to Figure 5: Production and consumption of metabolites by activated T cell subsets. Indicated T cell subsets were activated with anti-CD3/CD28 coated beads for 48 hours. **A.** Rates of consumption of selected amino acids and organic acids were determined by HPLC of supernatant and comparing that to un-used respective media kept at cell culture conditions for 48 hours. **B.** Rates of production of selected amino acids and organic acids were determined by HPLC of supernatant and comparing that to un-used respective media kept at cell culture conditions for 48 hours. nq= below limit of detection, not quantifiable. Rates of production are +/- SEM.

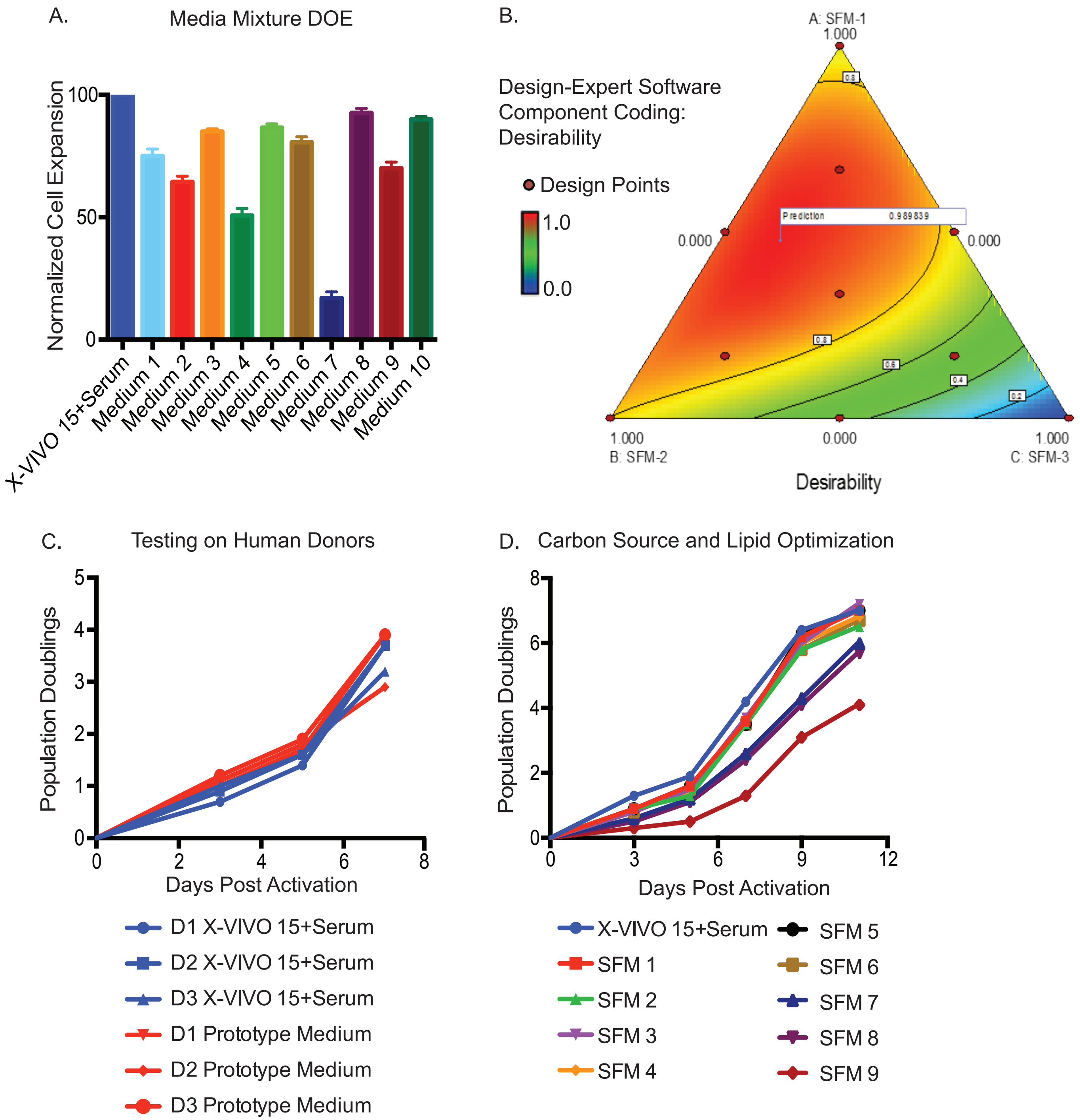

Ecker et al. Figure S1

Figure S1, related to Figure 1: Development and testing of IB2H media.

A. Primary human T cells were stimulated with anti-CD3/CD28 coated beads and cultured in one of ten custom media. Population doublings for each medium were normalized to the expansion obtained with X-VIVO™ 15 supplemented with 5% human AB serum. Data are representative from at least seven experiments. Error bars reflect SEM. These data were used for the statistical analysis to optimize cell expansion. **B**. Contour and response surface plots for designing the optimal media for T cell expansion. Statistical analysis performed on StatEase Design-Expert® 9.0.1 based on a Quadratic model with a desired response of maximum T cell serum-free expansion generated from results of prototype medias in A. Based on this analysis, a predicted optimal medium mixture formulation was generated. **C.** Total T cells were stimulated with anti-CD3/CD28 coated beads in X-VIVO™ 15 supplemented with 5% human AB serum or in the Design of Experiments (DOE) predicted medium (A). Data are representative of 3 donors. **D.** Total T cells were stimulated with anti-CD3/CD28 coated beads in X-VIVO™ 15 supplemented with 5% human AB serum or in the variants of the predicted medium containing a range of concentrations of glucose, galactose and lipids. Data are representative of 5 donors.

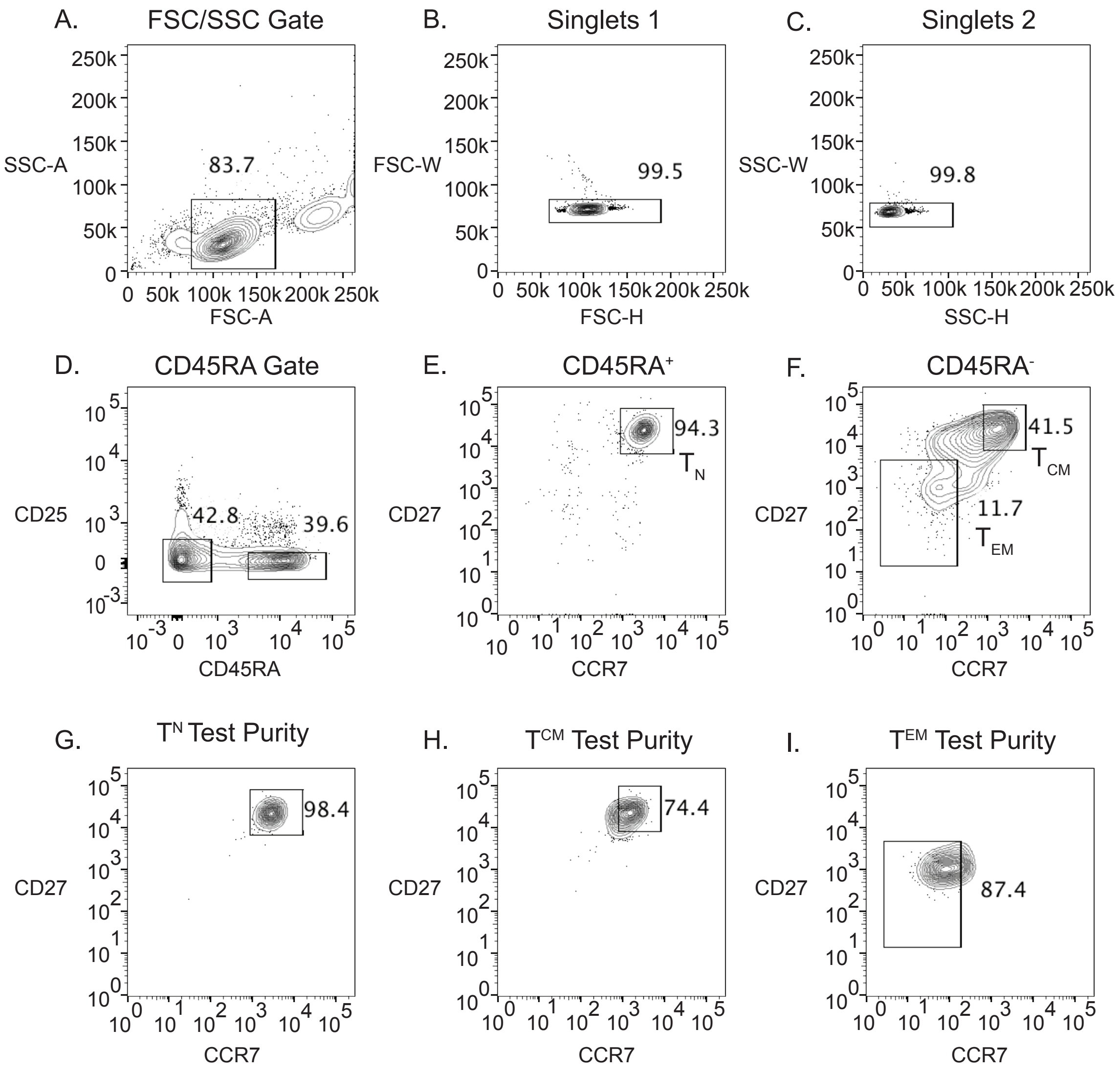

Ecker et al. Figure S2

Figure S2, related to Figure 1: Gating strategy and purities for sorting human naïve, central memory, and effector memory CD4 T cell subsets.

A-F. T cells for subset experiments were sorted using this gating strategy. Human CD4 T cells were isolated from peripheral blood mononuclear cells (PBMC) and obtained from the Human Immunology Core at the University of Pennsylvania. Cells were sorted in the following order by FSC-A/ SSC-A gating, singlet 1 gating, singlet 2 gating, CD45RA and CD25 gating, and then by CCR7 and CD27. **E.** Naïve (T_N) T cells were designated as cells that are $CD45RA^+$, $CD25^-$, $CCR7^+$, $CD27^+$. **F.** Central memory (T_{CM}) T cells were designated as cells that are $CD45RA^-$, $CD25^-$, $CCR7^+$, $CD27^+$. Effector memory (T_{EM}) T cells were designated as cells that are $CD45RA^-$, $CD25^-$, $CCR7^-$, $CD27^-$. **G-I**. Sorted populations were immediately re-run through the sorter to measure CCR7 and CD27 expression to determine sorting purity. Data is representative of dozens of sorts performed for publication.

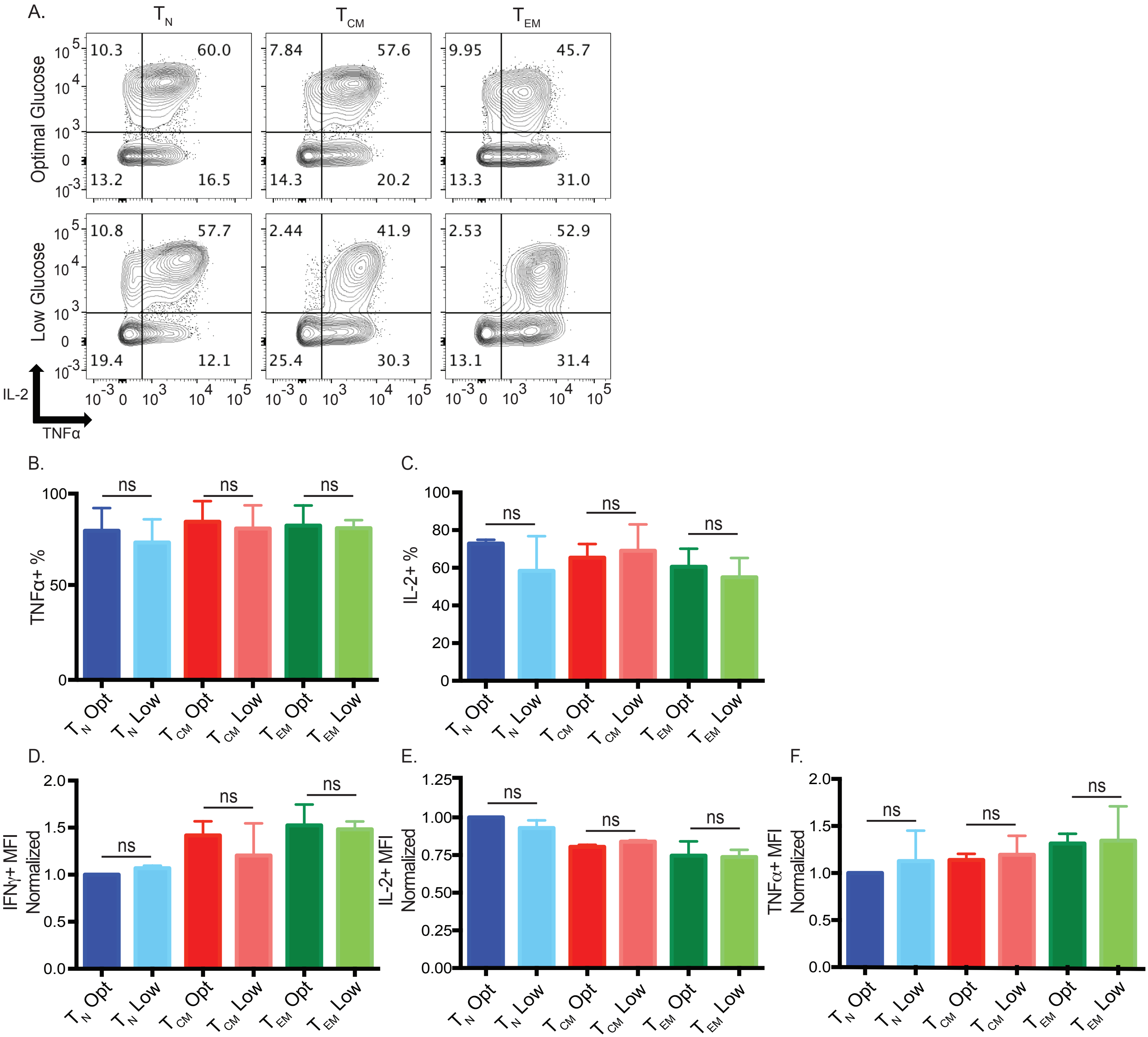

Ecker et al. Figure S3

Figure S3, related to Figure 2: IL-2 and TNFα production is not significantly affected by glucose in human CD4 T cells.

A. T cell subsets were activated as described in 2C and TNFα production was measured 9 days post-stimulation after PMA/ionomycin treatment. **B-C.** Percentages of TNFα and IL-2+ cells were quantified from A. **D-F.** Quantification of MFI from IFNy, IL-2, TNFα positive cells in optimal or low glucose 9 days post-stimulation after PMA/ionomycin treatment. Error bars reflect SEM. All data is representative of 3 independent experiments. *p <0.05, **p<0.01, paired two-tailed Student's T test. ns = not significant.

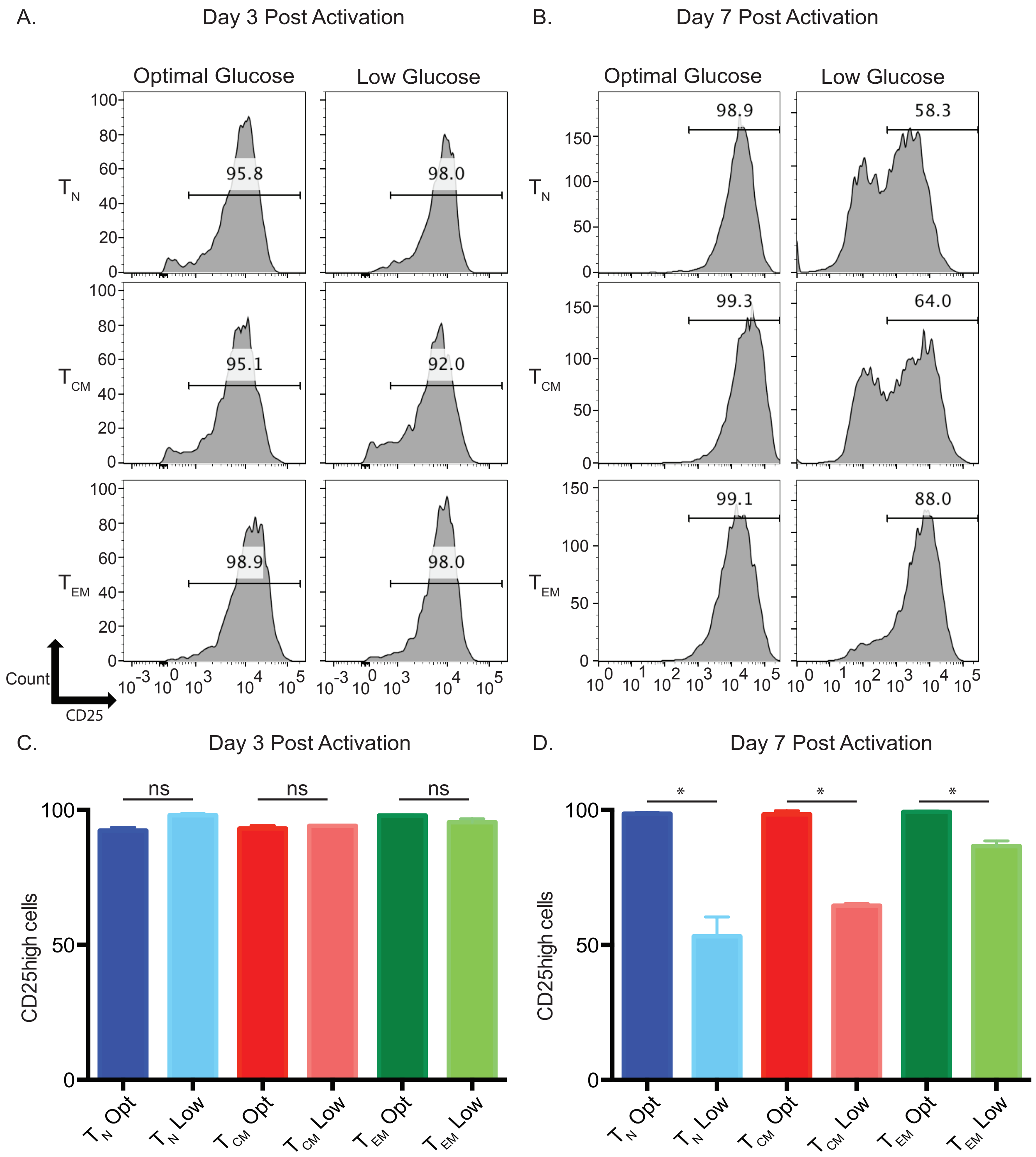

Ecker et al. Figure S4

Figure S4, related to Figure 2: Effector memory T cells express CD25 for a longer duration post-activation in limiting glucose.

A-B. Indicated T cell subsets were activated for 3 (A) or 7 days (B) using anti-CD3/CD28 coated beads and CD25 expression was measured. **C-D.** Percentage of CD25 high expressing cells on days 3 and 7 post-activation were quantified from A. and B. respectively. Error bars reflect SEM. Data are representative of 3 independent experiments. *$p < 0.05$, **$p < 0.01$, paired two-tailed Student's T test.

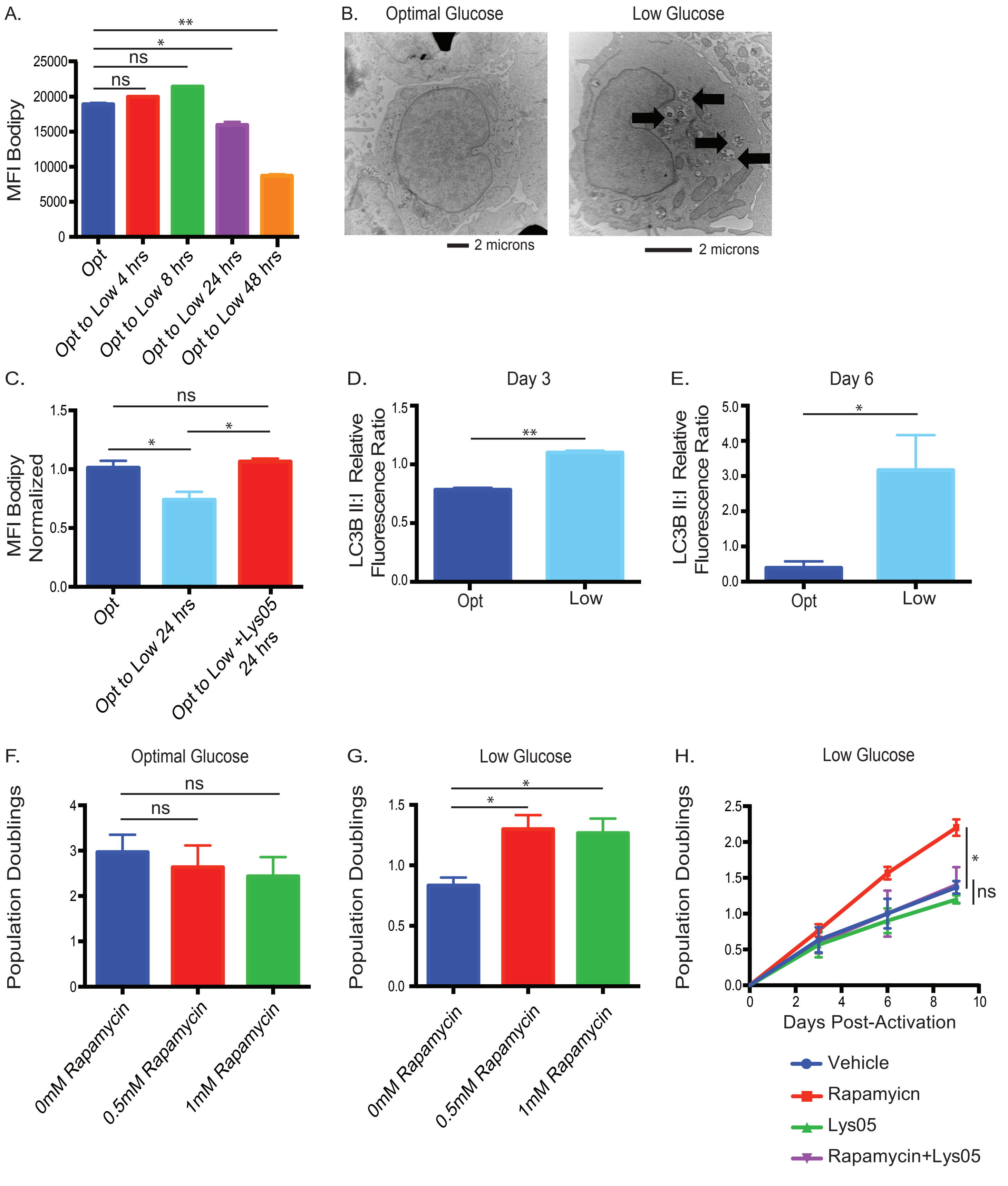

Ecker et al. Figure S5

Figure S5, related to Figure 4: Quantification of loss of lipid droplets and autophagy by activated T cells in limiting glucose.

A. Total CD4 T cells were activated as described in Figure 4B. Mean fluorescence intensity (MFI) was measured by flow cytometry. Error bars reflect SEM. Data are representative of 3 independent experiments. **B.** Total CD4 T cells were activated with anti-CD3/CD28 coated beads and placed in optimal or low glucose. After six days, T cells were examined using transmission electron microscopy. Arrows indicate presence of autophagosomes. **C.** Total CD4 T cells were activated with anti-CD3/CD28 coated beads in optimal glucose for 48 hours and then transferred into medium with low glucose or low glucose plus Lys05 for an additional 24 hours. Bodipy 493/503 was used to stain the cells at the indicated time points after being transferred to low glucose for 0, 4, 24, or 48 hours. **D-E.** Sorted T cells were activated with anti-CD3/CD28 coated beads for 3 or 6 days in optimal or low glucose. Cell lysates were probed for LC3B isoforms and β-actin. Western data is quantified via densitometry. **F-G.** Total CD4 T cells were activated with anti-CD3/CD28 coated beads for 5 days in optimal or low glucose in the presence or absence of rapamycin. Population doublings were quantified at day 5 post-activation. **H.** Total CD4 T cells were activated with anti-CD3/CD28 coated beads for 9 days in optimal or low glucose in the presence or absence of rapamycin, Lys05, or both rapamycin and Lys05. Data is representative of 3 independent experiments and donors. Error bars reflect SEM. $^*p < 0.05$, $^{**}p < 0.01$, paired two-tailed Student's T test or in case of multiple comparisons, one-way ANOVA followed by Tukey LSD**.** ns = not significant.

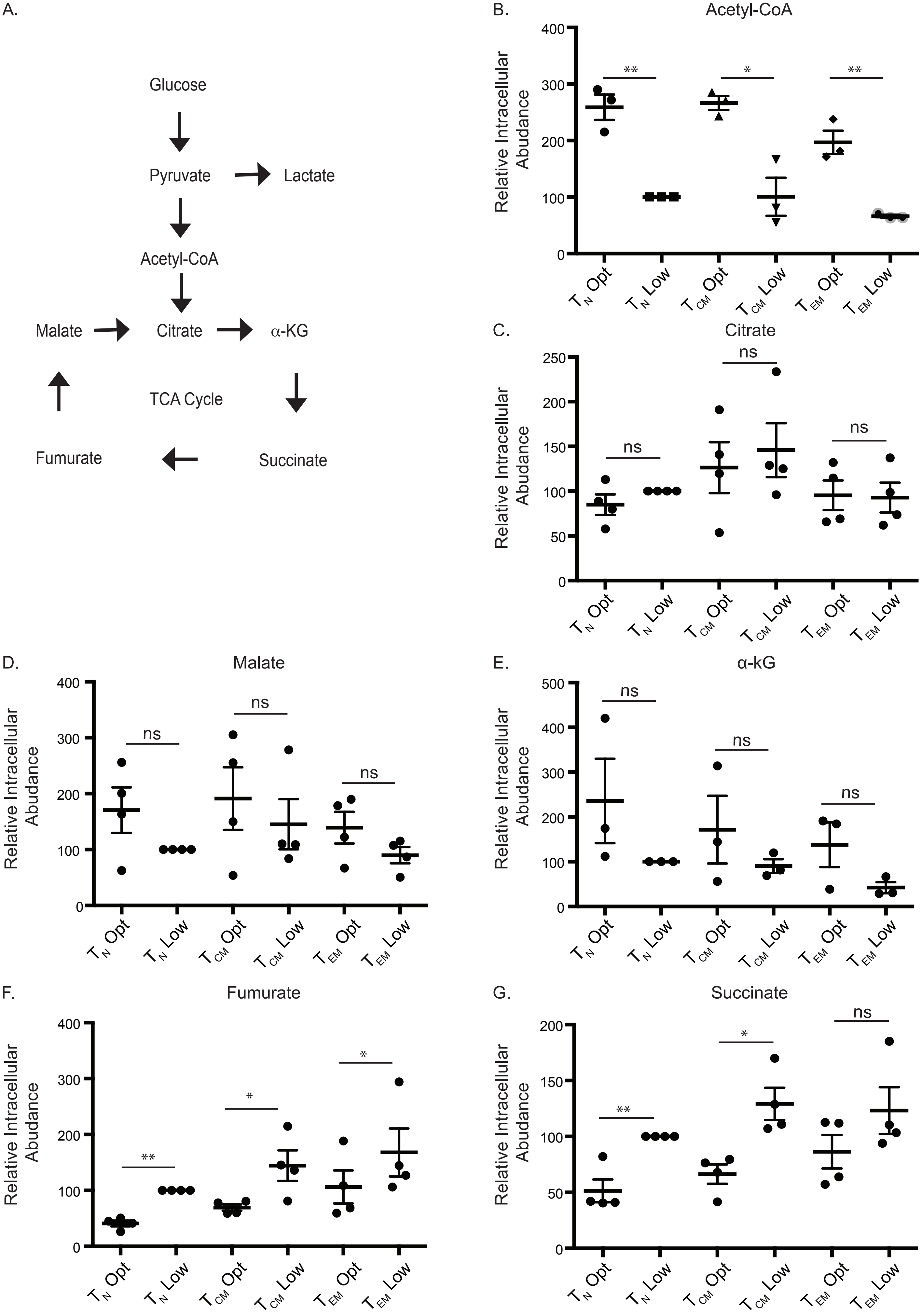

Ecker et al. Figure S6

Figure S6, related to Figure 5: Intracellular abundances of acetyl-CoA and TCA intermediates in activated T cell subsets.

A. Diagram depicting glycolysis and TCA cycle. **B-G.** Relative intracellular abundances of acetyl-CoA, citrate, malate, α-ketoglutarate (α-KG), fumurate, and succinate respectively from indicated subsets at 48 hours by LC-MS, normalized by cell number and cell volume. Error bars reflect SEM. All data is representative of 3-4 independent experiments. *p <0.05, **p<0.01, paired two-tailed Student's T test. ns = not significant.

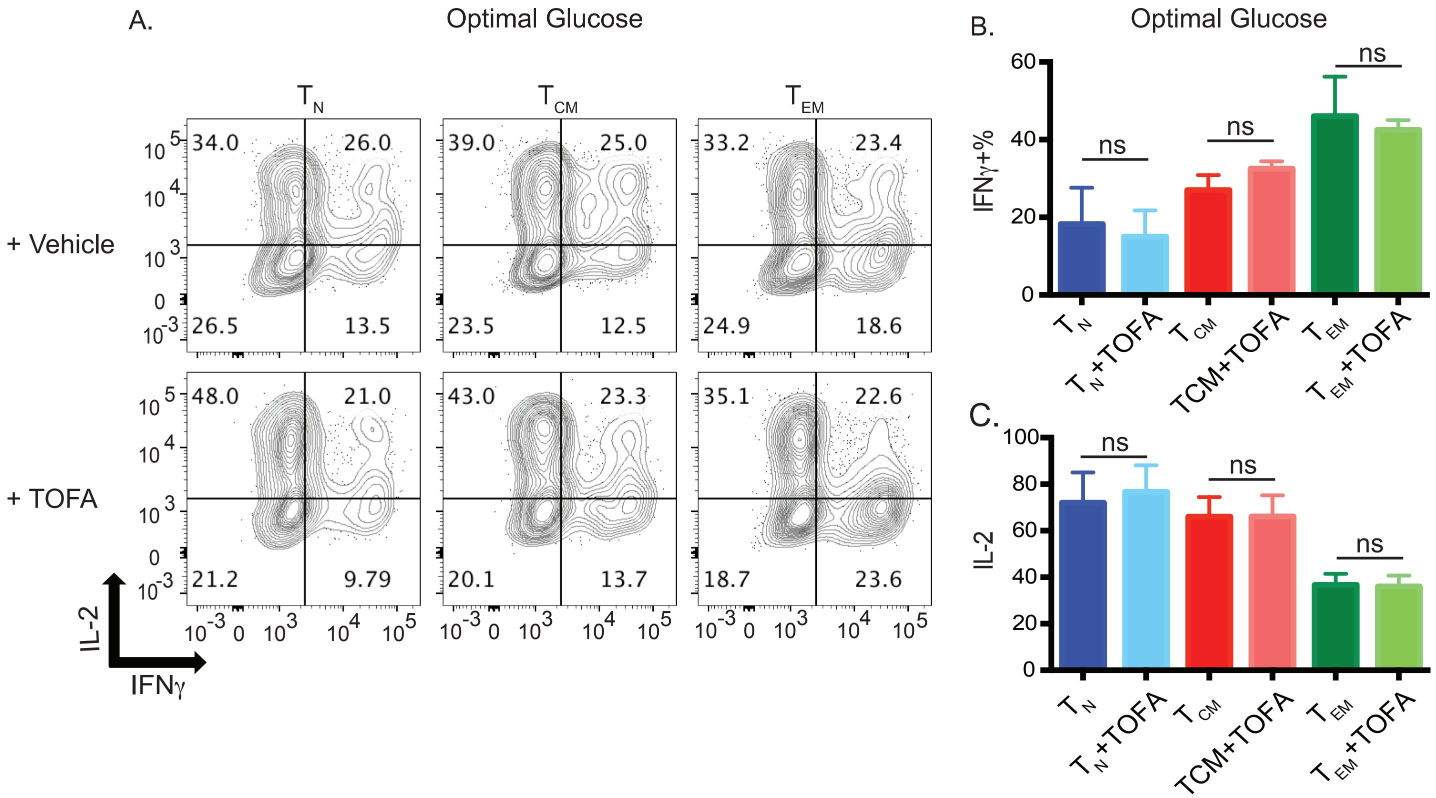

Ecker et al. Figure S7

Figure S7, related to Figure 7: Inhibition of fatty acid synthesis does not significantly affect cytokine production in optimal glucose.

A. Indicated subsets were activated as in Fig 6A and cytokine expression was measured after PMA/ionomycin treatment in optimal glucose. **B-C.** Data from A. quantified in 4 independent experiments. Error bars reflect SEM. *p <0.05, **p<0.01, paired two-tailed Student's T test. ns = not significant.

EXTENDED EXPERIMENTAL DATA

Cell Culture and Activation

Sorted CD4 T cells were washed twice in PBS, and then placed in IB2H serum-free medium containing optimal (35mM) or low (0.35mM) glucose concentrations. Cells were then activated at 1 million cells per mL using Dynabeads® Human T-Expander CD3/CD28 (ThermoFisher Scientific, 11131D) at a concentration of 3 beads per cell. Additional volumes of medium were added on Day 3 and every day after so that each culture was at 0.5 million cells/mL after feeding. Cells were treated with Lys05 (3uM, generously provided by Ravi Amaravadi) or rapamycin (500nM, SigmaAldrich, R0395) when indicated.

HPLC

Spent media analysis: Amino acids were quantified using a modified gradient UPLC method involving derivatization of amino acids, separation with a BEH C18 1.7µm Column with formate buffer/acetonitrile gradient followed by UV detection. The concentration of select water-soluble vitamins was determined using ion-pairing reverse phase chromatography with a C18 column followed by UV detection. The concentration of various organic acids (i.e. tricarboxylic acid cycle intermediates) was determined using ion exclusion column technology and detection via UV or RI. All analyses were performed by Gibco™ BioProduction Analytical Services.

Western Blotting

Cells were lysed with 1x RIPA Buffer (Cell Signaling Technology, 9806) and 1mM PMSF (Cell Signaling Technology, 8553S) according to manufacturer's instructions. Proteins were resolved by SDS-PAGE and transferred to nitrocellulose. Blots were probed with anti-LC3B (Cell Signaling Technology, 3868) and anti-β-actin (Cell Signaling Technology, 4970). Protein was visualized using Odyssey CLx LI-COR instrument. Densitometry was quantified using Image Studio 5.2 software.
